# Imaging the time course of DNA damage response at a nonrepetitive endogenous locus

**DOI:** 10.1016/j.crmeth.2025.101219

**Published:** 2025-11-03

**Authors:** Adam T. Rybczynski, W. Taylor Cottle, Po-Ta Chen, Jiwoong Kwon, Tiantian Shang, Yanbo Wang, Paul Meneses, Sushil Pangeni, Yeji Park, Momcilo Gavrilov, Taekjip Ha

**Affiliations:** 1 Howard Hughes Medical Institute and Program in Cellular and Molecular Medicine, Boston Children’s Hospital, Boston, MA, USA; 2 Department of Biology, Johns Hopkins University, Baltimore, MD, USA; 3 Department of Biophysics and Biophysical Chemistry, Johns Hopkins University School of Medicine, Baltimore, MD, USA; 4 Department of Biophysics, Johns Hopkins University, Baltimore, MD, USA; 5 Department of Pediatrics, Harvard Medical School, Boston, MA, USA

**Keywords:** DNA damage response, DNA repair, DNA DSB, DNA double-strand breaks, DNA damage response kinetics, GOLDFISH, vfCRISPR, DNA FISH

## Abstract

DNA double-strand breaks (DSBs) are among the most genotoxic lesions. Investigating the cellular dynamics of repair factors during DSB repair requires methodologies that preserve both spatial and temporal information. Here, we describe a method for tracking repair progression over time at any desired genomic locus by combining DSB induction on the seconds timescale (very fast CRISPR) and genomic labeling using local genome denaturation (genome oligopaint via local denaturation fluorescence *in situ* hybridization [GOLDFISH]). Through protocol optimization to retain repair signatures such as γH2AX, p53-binding protein 1 (53BP1), and BRCA1, we show that the kinetics of DSB foci formation at nonrepetitive endogenous loci can be measured with minutes time resolution.

## Highlights

• A method for imaging minute-scale DNA repair dynamics at nonrepetitive genomic loci
• A serial fixation approach preserves repair proteins and supports chromatin imaging
• Time courses of 53BP1, γH2AX, BRCA1, and chromatin decompaction

## Motivation

Repair of DNA double-strand breaks (DSBs) is central to understanding genome integrity, cellular survival, and disease mechanisms. Current approaches for studying DSB repair kinetics are constrained by limited genomic context, insufficient temporal control, or an inability to resolve cell-to-cell heterogeneity. To address these limitations, we developed a protocol that enables high spatiotemporal resolution of DNA repair processes at any site in the genome.

## Graphical abstract

**Figure:**
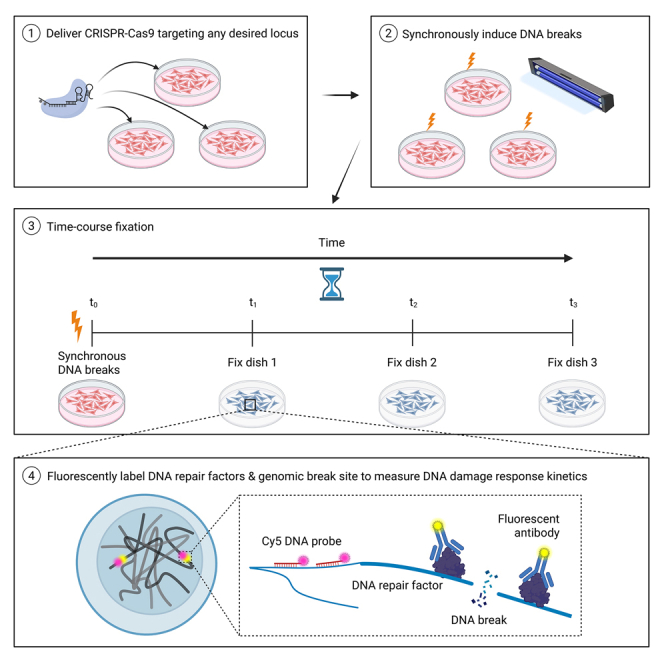

## Teaser

Rybczynski et al. introduce a method to measure DNA repair timing at specific genomic loci using very fast CRISPR and labeling of locally denatured genomic DNA and associated repair proteins. This approach enables detailed analysis of diverse repair processes, advancing our understanding of genomic instability and its role in disease.

## Introduction

DNA double-strand breaks (DSBs) represent one of the most severe forms of DNA damage. DSBs can arise from endogenous sources such as reactive oxygen species and cellular metabolism or from exogenous sources such as radiation and chemical mutagens.^1^ If improperly repaired, DSBs may lead to genomic instability, chromosomal aberrations, and potentially tumorigenesis or cell death.^2^ Repair of DSBs must occur across diverse chromatin contexts and can initiate within seconds of damage induction. Capturing the spatiotemporal dynamics of DSB repair requires methods that preserve spatial information relative to genome architecture while enabling synchronized, targeted, and time-controlled DNA damage induction.

CRISPR-associated protein 9 (Cas9) is a powerful genome-editing tool that has also been utilized to study DNA repair mechanisms.^3^ For DSB repair studies, Cas9 offers distinct advantages over earlier approaches such as nuclease- or radiation-induced damage, which exhibit low efficiency or produce delocalized, non-specific lesions. However, the lack of synchronous CRISPR activation on a sub-hour timescale has hindered investigations into the temporal order and kinetics of DNA repair processes. To address this, the very fast CRISPR (vfCRISPR) system was developed, enabling synchronous Cas9 activation with second-scale resolution.^4^ In this “light-on” approach, Cas9 is pre-bound to the target site through a caged guide RNA (gRNA). Upon light-mediated uncaging, the pre-bound Cas9 cleaves the DNA within seconds, offering a dramatic improvement in temporal control compared to prior arts. vfCRISPR has proven to be a powerful tool for dissecting DNA repair kinetics at DSB sites.^4^^,^^5^^,^^6^^,^^7^^,^^8^^,^^9^

Fluorescence microscopy can capture three-dimensional characteristics of DSB foci in single cells and has been widely used to study repair foci formation. It offers advantages over population-level methods such as chromatin immunoprecipitation sequencing (ChIP-seq), assay for transposase-accessible chromatin sequencing (ATAC-seq), and Hi-C sequencing in DSB repair studies by allowing the assessment of cell-to-cell heterogeneity and measurement of repair focus size. Live-cell imaging has been instrumental for investigating repair kinetics after irradiation- or nuclease-induced damage. Cas9 has been employed to label repetitive sequences for tracking repair foci dynamics, but labeling nonrepetitive sites remains challenging and typically requires cell fixation for either traditional DNA fluorescence *in situ* hybridization (FISH) or Cas9-mediated imaging approaches.^10^^,^^11^^,^^12^^,^^13^^,^^14^

Traditional DNA FISH involves global genome denaturation using heat or high concentrations of formamide. Although several non-denaturing FISH methods have been developed, they are generally limited to repetitive regions or lack the capacity to investigate DSB repair factor kinetics post damage.^15^^,^^16^ While live-cell techniques have emerged for imaging nonrepetitive loci^17^^,^^18^ and repair foci have been visualized using nanobodies for DNA repair-related histone marks,^19^ simultaneous observation of repair signals and specific genomic markers at nonrepetitive loci has not yet been achieved. Recently, genome oligopaint via local denaturation FISH (GOLDFISH) has emerged as a promising alternative.^20^ In GOLDFISH, a mutant Cas9, capable of cleaving only the non-target strand, is used as a programmable loader for the highly processive engineered superhelicase Rep-X.^21^ This system generates single-stranded regions for oligo probe hybridization without global denaturation.^20^ GOLDFISH supports labeling of nearly any genomic locus using short oligonucleotide probes that are readily synthesized and labeled^22^ and has even been shown to detect single-nucleotide polymorphism via cellular imaging.^23^ In principle, GOLDFISH better preserves chromatin ultrastructure by avoiding harsh denaturation protocols.

Combining vfCRISPR and GOLDFISH with immunofluorescence (IF) for DNA repair-associated proteins holds potential for investigating DNA repair at specific loci on a timescale that matches the rapidity of DSB repair. However, some repair epitopes used in IF are sensitive to the fixation conditions required for GOLDFISH as we demonstrate here. Most IF studies rely on crosslinking fixatives, which are compatible with traditional DNA FISH and widely used in the field. In contrast, Cas9-based labeling methods typically avoid crosslinking,^16^^,^^20^ likely due to fixation-induced hindrance of Cas9 DNA access. Instead, Cas9-mediated techniques use organic fixation under acidic conditions, which can extract DNA-binding proteins such as histones, making IF detection of certain epitopes challenging.^24^

In this study, we integrate vfCRISPR and GOLDFISH with IF for studying DNA DSB repair, by evaluating various fixation and labeling strategies to identify conditions that preserve fixation-sensitive DNA repair proteins. We developed a serial organic fixation protocol that enables robust IF alongside efficient GOLDFISH labeling. This approach, when combined with vfCRISPR, allowed us to characterize the accumulation kinetics of repair-associated markers at a defined genomic site. We anticipate that this IF-compatible GOLDFISH method will serve as a valuable tool for chromatin imaging, including studies of epigenetic histone modifications.

## Results

### *ACTB* E2 GOLDFISH labeling

**Figure 1:**
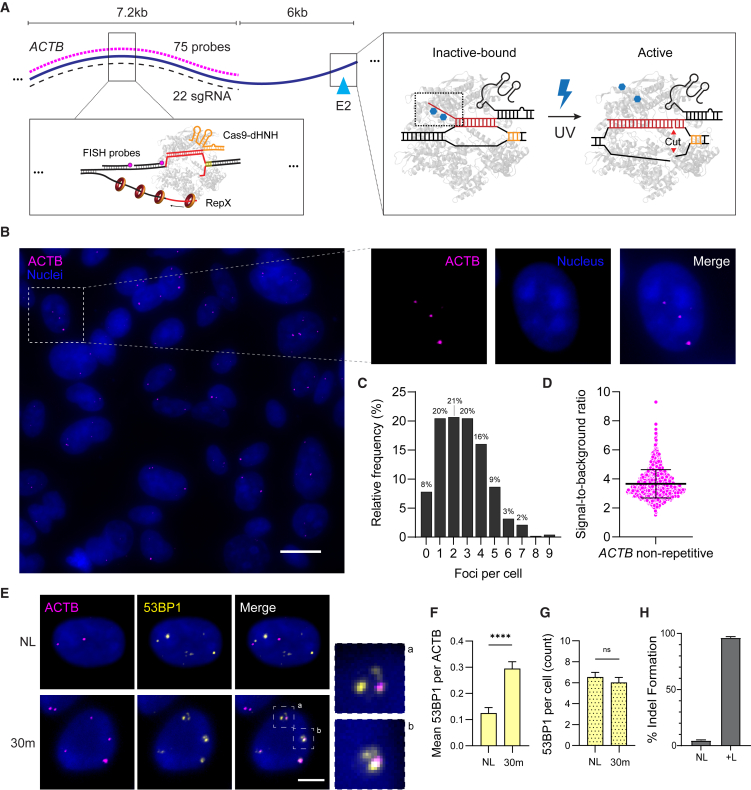
Combining vfCRISPR and GOLDFISH to study DNA damage response at a nonrepetitive genomic locus (A) Schematic of the experimental protocol. GOLDFISH (left): in fixed cells following vfCRISPR cleavage, Cas9 nickase targets a DNA strand at 22 sites across 7.2 kb. The superhelicase Rep-X unwinds DNA downstream starting from the 3′ end of the cleaved non-target strand. Fluorescently labeled FISH probes anneal to the exposed target strand. vfCRISPR (right): in live cells, Cas9 complexed with caged gRNA is pre-bound to the *ACTB* E2 site. UV light uncages the gRNA, inducing a DSB within seconds. (B) Representative GOLDFISH images showing *ACTB* signal (magenta) in U2OS cells; cutaway reveals a single cell. Scale bar: 15 μm. Nuclei stained with Hoechst (blue). (C) Histogram of GOLDFISH *ACTB* spots per cell (*n* = 2 replicates, ∼50 cells per replicate). (D) Signal-to-background ratio of *ACTB* foci (mean = 3.65). Bars represent mean ± SD from *n* = 3 replicates (∼100 cells each). (E) Representative images of 53BP1 IF and *ACTB* GOLDFISH at various time points post vfCRISPR activation in U2OS cells. Scale bar: 5 μm. Insets show zoomed-in merged images. (F) Mean ± SEM per cell of 53BP1 foci colocalized with *ACTB* spots at 30 min after vfCRISPR activation vs. no light (NL) control: NL = 0.13 ± 0.025, 30 min = 0.295 ± 0.03. *n* = 2 replicates (∼30 cells/condition). (G) Mean ± SEM of 53BP1 foci per cell at 30 min after vfCRISPR activation (6.0 ± 0.5) vs. NL control (6.5 ± 0.5). *n* = 2 replicates (∼30 cells/condition). (H) Indel percentage at the *ACTB* site 48 h after vfCRISPR activation vs. no-light control in U2OS cells. Bars represent mean indel percentage per electroporation: NL = 4.6%, (+)light = 96%. Error bars represent standard error: NL = 0.57, (+)light = 1.0. *n* = 4 electroporations. Statistical notation: ns indicates no significant shift. Statistical significance: *p* < 0.0001 (∗∗∗∗), determined by Kruskal-Wallis test. See also Table S1.

To visualize a nonrepetitive endogenous locus as a reference for studying nuclear features, we designed GOLDFISH labeling reagents targeting exon 2 of the β-actin (*ACTB* E2) gene, which we previously targeted using vfCRISPR to induce a single DSB.^4^ For GOLDFISH, gRNAs and probes were designed as previously described.^20^ To label the locus, we created 22 gRNAs and 75 fluorescently labeled probes targeting a 7.2 kb region located 6 kb upstream of the vfCRISPR DSB site (Figure 1A; Table S1). Using 22 gRNAs enabled Rep-X loading at multiple sites, mitigating potential obstacles during DNA unwinding or inefficient cleavage that could reduce labeling density. The GOLDFISH labeling region was positioned 6 kb upstream of the DSB to avoid interfering with DSB repair focus formation, while remaining within the diffraction limit of widefield microscopy to ensure colocalization of GOLDFISH and repair factor immunofluorescent signals. GOLDFISH showed high labeling efficiency in U2OS cells (Figure 1B), with an average of 3.4 *ACTB* foci per cell (Figure 1C). The lack of labeling in the absence of either Cas9 nickase or Rep-X confirmed that GOLDFISH spots are not due to non-specific probe binding (Figure S1). Additionally, the presence of more than two spots in many cells (Figure 1C) likely reflects the polyploid nature of U2OS cells. The signal-to-background (S/B) ratio of *ACTB* foci was sufficiently high to enable reliable detection (Figure 1D).

### 53BP1 recruitment time course at Cas9-mediated DSB

To study the DSB response at a specific endogenous locus, we applied vfCRISPR at *ACTB* E2. *ACTB* encodes β-actin and is constitutively expressed. At the same *ACTB* E2 site, we previously showed that more than half the sites are cleaved within seconds of light activation.^4^ Following illumination with 365 nm light to uncage the caged gRNA at the *ACTB* locus, we waited for 30 min before fixing the cells. We used IF to image p53-binding protein 1 (53BP1), a widely used marker of DSB repair, and performed GOLDFISH to fluorescently label *ACTB* sites (Figure 1E). Spot boundaries for each channel were identified by background subtraction followed by intensity thresholding. Each channel was then segmented into primary objects, 53BP1 and *ACTB*, based on these boundaries. The segmented objects were overlaid to determine the subset of 53BP1 foci that colocalized with *ACTB* sites (Figure S2).

We observed an increase in the number of 53BP1 foci colocalizing with *ACTB* spots following DSB induction (Figure 1F). In contrast, the total number of 53BP1 foci across the nucleus did not significantly change (Figure 1G), underscoring the importance of locus-specific labeling for accurately quantifying repair factor recruitment. Sanger sequencing confirmed indel formation exclusively in samples exposed to light (Figures 1H and S1).

### Sequential organic fixation retains acid-sensitive nuclear protein signal localization for IF-ready GOLDFISH

The DNA damage response involves an array of proteins that detect, signal, and repair DNA lesions.^25^ A key event in DSB signaling is the phosphorylation of the histone variant H2AX (γH2AX), which spreads across megabase regions flanking the DSB and facilitates the recruitment of additional repair factors such as MDC1 and 53BP1.^26^ In its original implementation, GOLDFISH was performed in cells fixed with a 1:1 methanol-acetic acid (MAA) solution.^20^ Acidic organic fixation, commonly used in metaphase chromosome spreads, is known to preserve chromatin morphology but also to extract positively charged nuclear proteins.^24^ Accordingly, acidic organic fixation was incompatible with immunofluorescent detection of several DNA damage markers, including γH2AX and BRCA1 (Figure S3).

**Figure 2:**
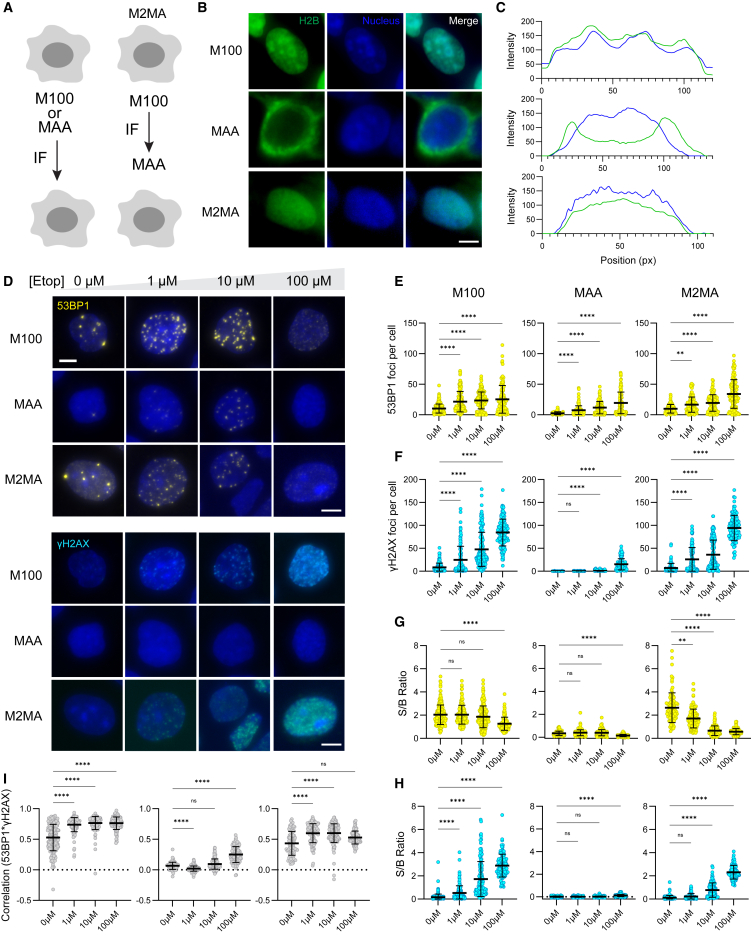
Sequential organic fixation preserves immunofluorescent labeling of weakly bound nuclear proteins (A) Schematic of fixation protocols for cells prior to immunofluorescence (IF). Methanol (M100) or 1:1 methanol:acetic acid (MAA) fixation (left), followed by IF. Sequential fixation (M2MA) (right): cells undergo M100 fixation, then IF, followed by MAA post fixation. (B) Representative IF images of H2B (green) across fixation strategies in HEK293T cells (top = M100, middle = MAA, bottom = M2MA). Nuclei stained with Hoechst (blue). Scale bar: 5 μm. (C) Representative intensity line plots corresponding to (B). Blue line: Hoechst intensity across a single cell. Green line: H2B intensity across a single cell. (D) IF images of U2OS cells across fixation conditions (top = M100, middle = MAA, bottom = M2MA) and etoposide treatments (left to right: 0, 1, 10, 100 μM). Top: 53BP1 (yellow); bottom : γH2AX (cyan). Hoechst stain (blue). Scale bars: 5 μm. (E) Scatterplots of 53BP1 foci per cell under different fixation conditions and etoposide concentrations: M100: 0, 1, 10, 100 μM → mean = 10, 22, 25, 28. MAA: 0, 1, 10, 100 μM → mean = 2, 8, 14, 22. M2MA: 0, 1, 10, 100 μM → mean = 10, 20, 23, 39. (F) Scatterplots of γH2AX foci per cell across fixation and etoposide conditions: M100: mean = 10, 25, 48, 92. MAA: mean = 0, 0, 5, 30. M2MA: mean = 8, 27, 40, 97. (G) Signal-to-background (S/B) ratio for 53BP1 foci: M100: mean = 2.0, 2.0, 1.9, 1.2. MAA: mean = 0.4, 0.4, 0.4, 0.1. M2MA: mean = 2.5, 1.8, 0.6, 0.5. (H) S/B ratio for γH2AX foci: M100: mean = 0.1, 0.6, 1.8, 3.2. MAA: mean = 0, 0, 0.1, 0.2. M2MA: mean = 0.1, 0.2, 0.9, 2.3. (I) Colocalization of 53BP1 and γH2AX foci (mean values): M100: 0.53, 0.76, 0.80, 0.80. MAA: 0.07, 0.01, 0.10, 0.29. M2MA: 0.45, 0.60, 0.60, 0.55. All data are presented as mean ± SD. ns indicates no significant shift. Statistical significance: *p* < 0.05 (∗), *p* < 0.01 (∗∗), *p* < 0.001 (∗∗∗), *p* < 0.0001 (∗∗∗∗), determined by Kruskal-Wallis test (*n* = 2 replicates, ∼50 cells per condition per replicate).

To improve compatibility between IF and GOLDFISH, we tested alternative organic fixatives and serial fixation strategies (Figure 2A), using histone H2B as a proxy for protein retention. In HEK293T cells, fixation with 100% methanol (M100), a neutral organic fixative, preserved nuclear H2B localization, whereas MAA yielded diffuse nuclear peripheral staining (Figures 2B and 2C, top and middle rows), suggesting histone loss from chromatin. To circumvent this limitation, we implemented a serial protocol, termed M100-to-MAA (M2MA), in which cells were first fixed with M100, followed by IF labeling of H2B, then a second fixation step with MAA. This strategy preserved H2B nuclear localization (Figures 2B and 2C, bottom row).

To assess whether M100, MAA, or M2MA fixation enabled immunofluorescent labeling of DNA repair-associated proteins, we treated cells with increasing concentrations of etoposide, a topoisomerase II inhibitor that prevents religation during S and G2 phases, resulting in DSB accumulation.^27^^,^^28^^,^^29^ Post treatment, we performed IF for 53BP1 and γH2AX in U2OS cells (Figure 2D). In M100-fixed cells, both 53BP1 (Figure 2E, left) and γH2AX (Figure 2F, left) foci were readily detected and increased in number with increasing etoposide concentration. Under MAA fixation, 53BP1 foci remained detectable (Figure 2E, middle), but γH2AX was only visible at the highest etoposide dose (Figure 2F, middle), consistent with histone extraction noted earlier. The M2MA protocol preserved 53BP1 and γH2AX foci and maintained their spatial localization (Figures 2E and 2F, right), indicating that initial M100 fixation allows labeling of histone-associated epitopes, and the subsequent MAA fixation does not interfere.

We next quantified the S/B ratios for IF labeling of 53BP1 and γH2AX across fixation methods in etoposide-treated cells. For 53BP1, S/B ratios were comparable between M100 and M2MA, while MAA showed a 10-fold reduction, confirming its suboptimal performance (Figure 2G). Of note, 53BP1 S/B ratio declined across all conditions at 100 μM etoposide, likely due to 53BP1 exhaustion.^30^ For γH2AX, M100 and M2MA produced robust signals, whereas MAA yielded no detectable fluorescence (Figure 2H). Because γH2AX is essential for 53BP1 recruitment,^1^ we examined colocalization between both markers under each fixation condition. M2MA closely reproduced the colocalization patterns observed in M100, supporting its utility for DSB marker analysis (Figure 2I).

Finally, we tested whether the M2MA sequential fixation protocol supports concurrent IF detection of 53BP1 and γH2AX alongside GOLDFISH labeling of the *ACTB* locus. Following DSB induction at *ACTB* via vfCRISPR, cells were first fixed in M100, labeled for 53BP1 and γH2AX via IF, then refixed in MAA, and subjected to GOLDFISH. All three signals—53BP1, γH2AX, and *ACTB*—were successfully detected (Figure S3). Thus, we identified a fixation strategy that preserves immunolabeling of fixation-sensitive nuclear proteins while enabling locus-specific detection via GOLDFISH.

### Time courses of γH2AX and 53BP1 accumulation at a single DSB site

**Figure 3:**
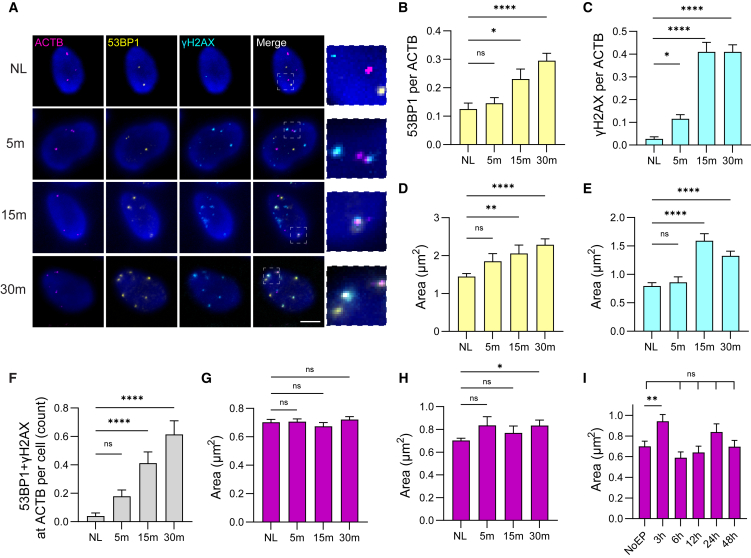
Localized recruitment kinetics and characterization of 53BP1 and γH2AX at a nonrepetitive endogenous locus (A) Representative images of *ACTB* GOLDFISH (magenta), 53BP1 IF (yellow), and γH2AX (cyan) at indicated time points following vfCRISPR activation in U2OS cells. Numbers on the left indicate time of fixation after vfCRISPR activation; NL, no light control; m, minutes post vfCRISPR activation. Nuclei stained with Hoechst (blue). Insets show zoomed-in merged views. Scale bar: 5 μm. (B) Quantification of 53BP1 foci per *ACTB* spot as a function of time after vfCRISPR activation: NL control = 0.13; 5 min = 0.15; 15 min = 0.25; 30 min = 0.30. (C) Quantification of γH2AX foci per *ACTB* spot: NL = 0.012; 5 min = 0.12; 15 min = 0.42; 30 min = 0.42. (D and E) Quantification of 53BP1 and γH2AX foci area per *ACTB* spot: 53BP1: NL = 1.5 μm^2^; 5 min = 1.9 μm^2^; 15 min = 2.1 μm^2^; 30 min = 2.4 μm^2^. γH2AX: NL = 0.8 μm^2^; 5 min = 0.9 μm^2^; 15 min = 1.6 μm^2^; 30 min = 1.4 μm^2^. (F) Quantification of *ACTB* spots containing both 53BP1 and γH2AX foci per cell: NL = 0.025; 5 min = 0.2; 15 min = 0.4; 30 min = 0.6. (G) Quantification of total *ACTB* spot area as a function of time: NL = 0.70 μm^2^; 5 min = 0.70 μm^2^; 15 min = 0.65 μm^2^; 30 min = 0.72 μm^2^. (H) Area of *ACTB* spots colocalized with both 53BP1 and γH2AX foci: NL = 0.70 μm^2^; 5 min = 0.82 μm^2^; 15 min = 0.78 μm^2^; 30 min = 0.82 μm^2^. (I) Total *ACTB* spot area following CRISPR-Cas9 electroporation targeting *ACTB* over a time course: NoEP (no electroporation) = 0.70 μm^2^; 3 h = 0.94 μm^2^; 6 h = 0.59 μm^2^; 12 h = 0.64 μm^2^; 24 h = 0.84 μm^2^; 48 h = 0.70 μm^2^. All values are presented as mean ± SEM. ns indicates no significant change. Statistical significance: *p* < 0.05 (∗), *p* < 0.01 (∗∗), *p* < 0.001 (∗∗∗), *p* < 0.0001 (∗∗∗∗), determined by Kruskal-Wallis test (*n* = 2 replicates, ∼30 cells per condition per replicate).

We next applied the M2MA fixation strategy to measure the time course of γH2AX and 53BP1 accumulation at 5, 15, and 30 min following DSB induction at the *ACTB* site via vfCRISPR (Figures 3A and S5). To quantify colocalization, we calculated the fraction of *ACTB* spots that overlapped with 53BP1 foci by dividing the number of colocalization events by the total number of *ACTB* spots. At 5 min, the colocalized fraction remained unchanged relative to the no-light control but increased by 2-fold and 2.5-fold at 15 and 30 min, respectively (Figure 3B), consistent with the ∼10 min recruitment kinetics of 53BP1 observed following Cas9-induced damage.^4^

We performed the same analysis for γH2AX, determining the fraction of *ACTB* spots that colocalized with γH2AX foci. This fraction increased 10-fold at 5 min and further increased to ∼35-fold at both 15 and 30 min relative to the no-light control, indicating that γH2AX signal propagation occurs within the first 5–15 min post DSB (Figure 3C). We showed previously that γH2AX foci counts do not increase at the level of 365 nm UV exposure used for vfCRISPR activation.^4^ The delayed accumulation of 53BP1 relative to γH2AX suggests that our approach enables reliable kinetic profiling of DSB repair factors.^31^

For cells containing γH2AX, 53BP1, and *ACTB* signals, we determined the fraction of *ACTB* spots colocalized with both γH2AX and 53BP1. Compared to the no-light control, this fraction did not change significantly at 5 min but increased by 16.1-fold and 24.8-fold at the 15 and 30 min time points, respectively (Figure 3F).

Are the repair foci, which increase in number over time, also increasing in size? To address this question, we measured the mean area of repair foci at the *ACTB* locus. The mean 53BP1 foci area remained unchanged at 5 min but increased by 30% and 53% at 15 and 30 min, respectively, relative to the no-light control (Figure 3D), possibly due to 53BP1 oligomerization at DSB sites^32^ or propagation of γH2AX. Similarly, γH2AX foci area increased at 15 and 30 min but not at 5 min (Figure 3E). The parallel size increases of γH2AX and 53BP1 are consistent with the known requirement of H2AX phosphorylation for H2A ubiquitination and subsequent 53BP1 recruitment.^31^^,^^33^^,^^34^

Altogether, the time-resolved changes in γH2AX and 53BP1 foci number and size align with previous reports, validating the M2MA sequential fixation strategy for analyzing DSB repair kinetics involving fixation-sensitive nuclear repair factors at specific genomic loci.

### Time course of chromatin decompaction at a single DSB site

We next measured the area of GOLDFISH-labeled *ACTB* spots at 5, 15, and 30 min following DSB induction at the *ACTB* locus via vfCRISPR, an analysis that can serve as a proxy for chromatin decompaction after DNA damage (Figure S4). Although the mean *ACTB* foci area did not significantly vary across time points (Figure 3G), the subset of *ACTB* foci colocalized with both γH2AX and 53BP1 showed a 20% increase in area at the 30-min time point compared to the no-light control (Figure 3H), supporting chromatin decompaction following sustained DNA damage.^35^^,^^36^^,^^37^

In a separate experiment, cells were electroporated with Cas9 and regular gRNA targeting *ACTB* to observe repair dynamics over an extended period, between 3 and 48 h. *ACTB* foci size increased by 34% at the 3 h time point relative to non-electroporated controls, further indicating chromatin decompaction at the DSB site (Figure 3I). At later time points, *ACTB* spot size returned to levels comparable to undamaged controls, suggesting that repair of the initial DSB may occur as early as 6 h post induction. However, 53BP1 and BRCA1 foci remained detectable at the *ACTB* site throughout the 48 h time course, consistent with repeated Cas9-mediated cleavage events (Figure S4). We infer that prolonged Cas9 activity leads to asynchronous DSB induction at *ACTB*, resulting in heterogeneous repair timing across the cell population. This variability may explain why *ACTB* spot sizes at later time points are not statistically distinguishable from undamaged cells (Figure 3I).

### Time courses of BRCA1 and 53BP1 accumulation at a single DSB site

**Figure 4:**
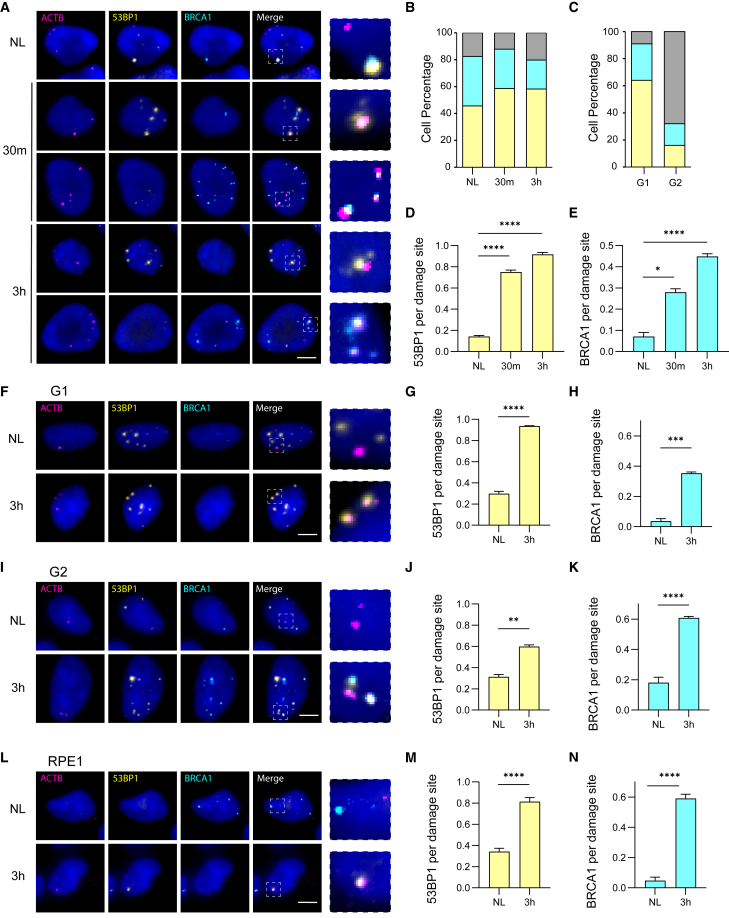
Localized recruitment kinetics and characterization of 53BP1 and BRCA1 at a nonrepetitive endogenous locus (A) Representative images of *ACTB* GOLDFISH (magenta), 53BP1 (yellow), and BRCA1 (cyan) IF at various time points following light-induced vfCRISPR activation in freely cycling U2OS cells. Hoechst (blue) marks nuclei. Insets show zoomed-in merged images. Scale bar: 5 μm. (B) Percentages of freely cycling U2OS cells exhibiting 53BP1 foci only (yellow), BRCA1 foci only (cyan), or both (gray) over time after vfCRISPR activation. For the no-light (NL) control: 53BP1 only, 46%; BRCA1 only, 37%; both, 17%. At 30 min (30m): 53BP1 only, 59%; BRCA1 only, 29%; both, 12%. At 3 h: 53BP1 only, 58%; BRCA1 only, 22%; both, 20%. (C) Percentages of U2OS cells synchronized in G1 or S/G2 phases displaying 53BP1 foci only (yellow), BRCA1 foci only (cyan), or both (gray). In G1: 53BP1 only, 64%; BRCA1 only, 27%; both, 9%. In S/G2: 53BP1 only, 16%; BRCA1 only, 16%; both, 68%. (D) Proportion of freely cycling U2OS cells showing colocalization of 53BP1 foci with *ACTB* among those containing both *ACTB* and 53BP1 foci. NL = 0.14; 30 min = 0.75; 3 h = 0.92. (E) Proportion of freely cycling U2OS cells showing colocalization of BRCA1 foci with *ACTB* among those containing both *ACTB* and BRCA1 foci. NL = 0.07; 30 min = 0.28; 3 h = 0.45. (F) Representative images of *ACTB* GOLDFISH (magenta), 53BP1 (yellow), and BRCA1 (cyan) IF at time points post vfCRISPR activation in G1-synchronized U2OS cells. Hoechst (blue) marks nuclei. Insets show merged closeups. Scale bar: 5 μm. (G) Colocalization of 53BP1 foci with *ACTB* in G1-synchronized cells as described in (D). NL = 0.3; 3 h = 0.94. (H) Colocalization of BRCA1 foci with *ACTB* in G1-synchronized cells as described in (E). NL = 0.04; 3 h = 0.35. (I) Representative images of *ACTB* GOLDFISH (magenta), 53BP1 (yellow), and BRCA1 (cyan) IF at time points post vfCRISPR activation in S/G2-synchronized U2OS cells. Hoechst (blue) marks nuclei. Insets show merged closeups. Scale bar: 5 μm. (J) Colocalization of 53BP1 foci with *ACTB* in S/G2-synchronized cells as described in (D). NL = 0.31; 3 h = 0.6. (K) Colocalization of BRCA1 foci with *ACTB* in S/G2-synchronized cells as described in (E). NL = 0.18; 3 h = 0.61. (L) Representative images of *ACTB* GOLDFISH (magenta), 53BP1 (yellow), and BRCA1 (cyan) IF at time points post vfCRISPR activation in freely cycling RPE1 cells. Hoechst (blue) marks nuclei. Insets show merged closeups. Scale bar: 5 μm. (M) Colocalization of 53BP1 foci with *ACTB* in RPE1 cells as described in (D). NL = 0.34; 3 h = 0.81. (N) Colocalization of BRCA1 foci with *ACTB* in RPE1 cells as described in (E). NL = 0.04; 3 h = 0.59. All data presented as mean ± SEM. Statistical significance: *p* < 0.05 (∗), *p* < 0.01 (∗∗), *p* < 0.001 (∗∗∗), *p* < 0.0001 (∗∗∗∗), determined by Kruskal-Wallis test (*n* = 2 replicates, ∼30 cells per condition per replicate).

The two principal pathways for repairing DSBs are non-homologous end joining (NHEJ) and homologous recombination (HR).^1^ NHEJ directly ligates broken DNA ends with minimal processing and is considered a relatively faithful repair mechanism, though repeated Cas9 cleavage can lead to indel formation. HR, by contrast, achieves high-fidelity repair using a sister chromatid with identical sequence as a template. Because HR requires sister chromatid availability, it predominantly occurs during the replicative (S) or post-replicative (G2) phases of the cell cycle. 53BP1 and BRCA1 are key mediators of DSB pathway choice: 53BP1 promotes NHEJ, whereas BRCA1 facilitates HR. We examined their recruitment to DSBs induced at the *ACTB* locus at 30 min and 3 h post DSB induction (Figures 4A and S5). We observed cell-to-cell heterogeneity in 53BP1 and BRCA1 foci, with some cells displaying one marker, both, or neither, likely reflecting cell cycle differences in the unsynchronized population (Figure 4B). Indeed, when we synchronized cells in G1 and S/G2 phases, G1 cells predominantly showed 53BP1 foci, while G2 cells exhibited an increased number of BRCA1 foci (Figure 4C).

For cells with 53BP1 foci and *ACTB* spots, we calculated the fraction of cells harboring damage foci at *ACTB* by dividing the number of cells with at least one colocalization event between 53BP1 and *ACTB* by the total number of cells positive for both markers. Compared to the no-light control, *ACTB* sites colocalizing with 53BP1 increased by 5.3-fold at 30 min and 6.6-fold at 3 h (Figure 4D). Using an equivalent analysis for BRCA1, the fraction of *ACTB* sites colocalizing with BRCA1 increased 4-fold and 6.4-fold at the 30 min and 3 h time points, respectively (Figure 4E).

To further validate the method, we synchronized cells using a double- or single-thymidine block, electroporated vfCRISPR, and induced DSBs at *ACTB* in either G1 or late S/G2 (Figures 4F, 4I, S5, and S6). G1 cells showed 53BP1 recruitment to *ACTB* sites with kinetics comparable to freely cycling cells at 3 h (Figure 4G), but BRCA1 recruitment was markedly reduced, consistent with its known restriction to late S/G2 phases (Figure 4H). In our experiments, 70% of cells were in G1 at the time of vfCRISPR-induced damage and 51% remained in G1 at the 3 h time point (Figure S6); the remaining BRCA1 recruitment in G1-synchronized cells, although much reduced compared to unsynchronized cells, suggests that residual HR activity may arise from cells not fully synchronized. In late S/G2 cells (Figure 4I), 53BP1 recruitment to *ACTB* decreased after 3 h relative to freely cycling cells (Figure 4J), while BRCA1 colocalization increased by 30%, consistent with enhanced HR initiation in G2 (Figure 4K).

We also applied our protocol to a human retinal pigment epithelial (RPE1) cell line, commonly used for DSB repair studies due to its normal karyotype^38^^,^^39^^,^^40^ (Figures 4L and S7). The same DSB induction and labeling workflow was used, and 53BP1 and BRCA1 recruitment to *ACTB* sites mirrored that observed in freely cycling U2OS cells (Figures 4M and 4N). Additionally, we targeted a second nonrepetitive endogenous locus, *MUC4*, in U2OS cells (Figure S8). Cells were fixed 3 h post electroporation with Cas9 and regular gRNA and compared to untreated controls. Recruitment of 53BP1 and BRCA1 to *MUC4* damage sites resembled their localization at *ACTB* in both U2OS and RPE1 cells (Figure S8). These experiments demonstrate the robustness of our workflow for other cell types and genomic locations.

## Discussion

DNA repair studies greatly benefit from technologies capable of introducing chemically well-defined damage at specific genomic loci. CRISPR-Cas9 meets this challenge by inducing a DSB at a location programmable via the gRNA sequence. vfCRISPR adds temporal control by synchronously activating pre-bound Cas9 within seconds of light exposure and has been combined with sequencing-based tools such as time-resolved ChIP-seq, ATAC-seq, and Hi-C to reveal the time course of repair factor accumulation and chromatin changes during repair.^4^^,^^5^^,^^6^^,^^9^ vfCRISPR has also been used with a repair reporter (53BP1-mCherry) in live-cell imaging; however, due to endogenous DNA damage unrelated to Cas9 activity, a nearby repetitive sequence was required for fluorescent labeling to attribute observed repair signals specifically to Cas9-induced damage.^4^

Here, we explored the possibility of studying DSB repair kinetics at arbitrary genomic loci, which may not be proximal to repetitive elements. To this end, we used DNA FISH to label regions near Cas9 target sites. Specifically, the recently developed GOLDFISH technique enables labeling of nonrepetitive sites with straightforward oligonucleotide probe design and minimal perturbation.^20^ vfCRISPR followed by GOLDFISH imaging of the target locus, combined with IF detection of repair factors, offers a powerful framework for visualizing repair kinetics. However, we found that the original GOLDFISH fixation protocol led to loss of nuclear proteins. To address this, we developed a sequential fixation workflow (methanol fixation → IF → MAA fixation → GOLDFISH) that preserves repair factor localization while remaining compatible with GOLDFISH.

We hypothesize that primary methanol fixation preserves protein-DNA interactions and nuclear localization, enabling IF detection. The secondary MAA step likely solubilizes basic proteins, rendering chromatin more accessible to Cas9 nickase and Rep-X required for GOLDFISH, while retaining previously bound antibodies.

We analyzed the area of *ACTB* foci underlying damage and observed a modest but significant ∼20% increase in the size of *ACTB* foci colocalized with 53BP1 and γH2AX after 30 min. This may reflect chromatin decompaction surrounding the DSB, a phenomenon previously observed following irradiation-induced damage, though it remains unclear whether this occurs at individual breaks or primarily at clustered lesions.^41^^,^^42^^,^^43^ Our extended time course showed further decompaction at 3 h post DSB induction, with potential recompaction over the subsequent 48 h. Decompaction has been monitored via sequencing after Cas9 cleavage, and accessibility changes attributed to nucleosome loss have been reported within 1 kb of cut sites following vfCRISPR-mediated damage. Since our GOLDFISH probes span ∼7 kb and are positioned ∼6 kb upstream of the cut site, the observed increase in foci area is unlikely to reflect proximal accessibility changes. The prevailing model for chromatin response to DNA damage—“access, repair, restore”—suggests nucleosome remodeling and local decompaction.^44^^,^^45^^,^^46^ Future studies using photocleavable RNAs to prevent repeated Cas9 cleavage^47^ could provide clearer insights into decompaction and recompaction dynamics.

NHEJ and HR are the major DSB repair pathways responsible for direct end joining with minimal processing and templated repair via DNA resection, respectively.^1^^,^^2^^,^^25^^,^^48^^,^^49^ NHEJ, initiated by Ku70/80, is active throughout the cell cycle and often mediates the earliest response to damage.^48^ HR occurs when the DSB is resected and is predominantly functional during S and G2 phases, when sister chromatids are available as templates.^50^ 53BP1 and BRCA1 modulate the activity of one another, controlling the timing and extent of resection by MRE11 and other nucleases.^51^^,^^52^ Here, we assessed the recruitment of 53BP1 and BRCA1 to Cas9-induced DSBs in freely cycling, G1-synchronized, and S/G2-synchronized cells. We generally observed three populations: cells with 53BP1 foci alone, BRCA1 foci alone, or both. The marked increase in BRCA1 foci in S/G2 vs. G1 is consistent with peak HR activity during mid-S and G2.^50^ Though BRCA1 is expressed across the cell cycle, its inability to form foci in G1 is likely regulated by 53BP1.^53^

These findings preview the potential of combining vfCRISPR, IF, and GOLDFISH for DNA repair research. With vfCRISPR, we can induce damage at any genomic site with temporal precision. Its light-on mechanism enables DSB induction in synchronized cells at defined cell cycle stages.^9^ Upon fixation at specified time points, repair proteins can be labeled using IF, potentially with multiplexing approaches,^54^ to interrogate repair foci formation. GOLDFISH enables locus-specific labeling via local DNA denaturation, preserving nuclear ultrastructure relevant to repair, and allows visualization of chromatin decompaction at the damage site. We demonstrated the versatility of our approach by applying it to a second nonrepetitive locus, *MUC4*, and to the *ACTB* site in RPE1 cells, underscoring its adaptability across loci and cell types. Because DSB repair involves a diverse repertoire of factors recruited and activated at the damage site, our platform provides a powerful window into their spatial and temporal interplay.

### Limitations of the study

Cross-linking fixation methods, including formaldehyde-based fixation, are commonly used to stabilize protein-DNA interactions and preserve chromatin architecture more robustly. The lack of cross-linking in our present method may limit the resolution or fidelity of protein localization and chromatin structure assessments, particularly for transient or weak interactions.

Another limitation is that vfCRISPR has not yet been validated in primary cells, which often exhibit distinct chromatin landscapes, DNA repair dynamics, and delivery challenges compared to immortalized cell lines.

Additionally, vfCRISPR requires light activation and efficient delivery of Cas9 components, which pose significant hurdles in tissue contexts. The current study does not address delivery mechanisms suitable for intact tissues or *in vivo* systems, thereby precluding kinetic studies of DNA repair in more complex biological environments.

These limitations highlight important avenues for future development, including optimization of fixation protocols, adaptation for primary cell use, and engineering of delivery strategies for tissue-level applications.

## Resource availability

### Lead contact

Further information and requests for resources and reagents should be directed to and will be fulfilled by the lead contact, Taekjip Ha (taekjip.ha@childrens.harvard.edu).

### Materials availability

This study did not generate new unique reagents.

### Data and code availability

• Raw imaging data can be accessed on Mendeley Data (https://doi.org/10.17632/3z3rft4hn2.1). The raw imaging data are multi-frame files, and the brightness/contrast may need to be adjusted to achieve optimal visualization. This study utilized the GRCh38.p13 primary assembly, obtained from the NCBI reference genome database.
• This study does not report original code.
• Any additional information required to re-analyze the data reported in this paper is available from the lead contact upon request.

## Acknowledgments

The authors thank Prof. Xin Chen (Johns Hopkins University), Prof. Bin Wu (Johns Hopkins School of Medicine), Prof. Yang Liu (University of Utah School of Medicine), Alberto Marin-Gonzalez (Harvard Medical School), and Sergei Rudnizky (Harvard Medical School) for their stimulating conversations throughout the project. The graphical abstract was created in BioRender (https://BioRender.com/mwupnw1). This project was supported by the 10.13039/100000002National Institutes of Health grants U01 DK127432 and R35 GM122569 and NSF Science and Technology Center for Quantitative Cell Biology (DBI 2243257) to T.H..

## Author contributions

W.T.C. and T.H. conceived the project. A.T.R. and W.T.C. designed and performed experiments and wrote the manuscript with contributions from all authors. A.T.R. and W.T.C. analyzed data and prepared figures. P.-T.C. made oligo probes for *ACTB* and *MUC4*. J.K. and Y.P. purified Cas9. J.K. validated *MUC4* gRNA. Y.W. assisted with conceptualization of the project. P.M. assisted with GOLDFISH experiments. P.-T.C. and T.S. purified Cas9 nickase. P.-T.C., T.S., S.P., and M.G. purified and crosslinked Rep-X. T.H. supervised the project and revised the manuscript.

## Declaration of interests

T.H. is a member of the editorial board of *Cell Reports Methods*.

## Declaration of generative AI and AI-assisted technologies in the writing process

During the preparation of this work, the authors used Microsoft Copilot in order to proofread the document. After using this tool, the authors reviewed and edited the content as needed and take full responsibility for the content of the publication.

## STAR★Methods

### Key resources table

**Table:** 

REAGENT or RESOURCE	SOURCE	IDENTIFIER
**Antibodies**		

Anti-53BP1	Novus Biologicals	Cat# NB100-304SS
Anti-H2B	Abcam	Cat# ab1790
Anti-γH2AX	Abcam	Cat# ab26350
Anti-BRCA1	Santa Cruz Biotechnology	Cat# sc-6954
Goat Anti-Mouse, Alexa Fluor 488	Thermo Fisher Scintific	Cat# A11001
Goat Anti-Rabbit, Alexa Fluor 555	Abcam	Cat# ab150078

**Bacterial and virus strains**		

BL21(DE3) Chemically Competent Cells	Sigma-Aldrich	Cat# CMC0014

**Chemicals, peptides, and recombinant proteins**		

Alt-R S.p. Cas9 Nuclease V3	IDT	Cat# 1081058
Alt-R S.p. Cas9 H840A Nickase V3	IDT	Cat# 1081064
Rep-X	Arslan et al.^21^	N/A
RNase Cocktail Enzyme Mix	Invitrogen	Cat# AM2286
Hoechst 33342 Ready Flow Reagent	Invitrogen	Cat# R37165
Cy5 NHS Ester	Sigma Aldrich	Cat# GEPA15101
Amino-11-ddUTP	Lumiprobe	Cat# A5040
Terminal Deoxynucleotidyl Transferase	Thermo Scientific	Cat# EP0162

**Critical commercial assays**		

SE Cell Line 4D-Nucleofector X Kit	Lonza	Cat# V4XC1032
P3 Primary Cell 4D-Nucleofector X Kit	Lonza	Cat# V4XP3032
DNeasy Blood & Tissue Kit	Qiagen	Cat# 69504
Q5 High-Fidelity 2× Master Mix	NEB	Cat# M0492L
GeneJet PCR Cleanup Kit	Thermo Fisher Scientific	Cat# K0701
EnGen RNA Synthesis	NEB	Cat# E3322V
Monarch Spin RNA Cleanup Kit	NEB	Cat# T2040

**Deposited data**		

Raw images	This paper; Mendeley Data	https://doi.org/10.17632/3z3rft4hn2.1

**Experimental models: Cell lines**		

U2OS	ATCC	Cat# HTB-96
HEK293T	ATCC	Cat# CRL-3216
RPE1	Laboratory of David Pellman	N/A

**Oligonucleotides**		

Alt-R CRISPR-Cas9 *ACTB* crRNA	IDT	N/A
Alt-R CRISPR-Cas9 *MUC4* crRNA	IDT	N/A
*ACTB* caged crRNA	Bio-Synthesis	N/A
Alt-R CRISPR-Cas9 tracrRNA	IDT	Cat# 1072533
See Table S1 for sequences of RNA oligonucleotides used in vfCRISPR experiments	Liu et al.^4^; This paper	N/A
See Table S1 for sequences of DNA oligonucleotides used for PCR	Liu et al.^4^; This paper	N/A
See Table S1 for sequences of DNA oligonucleotides used in GOLDFISH experiments	This paper	N/A

**Recombinant DNA**		

rep (C18L/C43S/C167V/C612A/S400C)	Arslan et al.^21^	N/A

**Software and algorithms**		

Fiji/ImageJ	Schneider et al.^55^	https://imagej.net/Fiji
CellProfiler	CellProfiler	https://cellprofiler.org/
NIS-Elements AR	Nikon	https://www.microscope.healthcare.nikon.com/products/software/nis-elements/
Oligoarray 2.1	Rouillard et al.^56^	https://che.engin.umich.edu/people/rouillard-jean-marie/
TIDE	Brinkman and van Steensel^57^	http://shinyapps.datacurators.nl/tide/

### Experimental model details

#### Cell lines and culture conditions

U2OS cells (ATCC, HTB-96) and HEK293T cells (ATCC, CRL-3216) were obtained from the American Type Culture Collection and cultured in DMEM (Corning, 10-013-CV) supplemented with 10% fetal bovine serum (FBS; Corning, 35-011-CV) and 1× antibiotic-antimycotic (AA; Gibco, 15240062). Human retinal pigment epithelial (RPE1) cells were generously provided by the laboratory of David Pellman and cultured in DMEM/F-12 (Gibco, 11320033) supplemented with 10% FBS and 1× AA. All cells were maintained at 37°C with 5% CO_2_ in a humidified incubator. Imaging dishes were coated with 1 μg/cm^2^ collagen prior to cell plating.

Cells were passaged using 0.05% trypsin/EDTA (Thermo Fisher Scientific, 25300120) every 2-4 days to maintain confluency below 90%. Mycoplasma contamination was routinely monitored using the Myco-Alert assay (Lonza).

### Method details

#### Cell fixation

M100 Fixation: 100% methanol was prechilled to −20°C. Cells were fixed in the chilled methanol for 5 min at −20°C, followed by three washes with PBS. Samples were stored at 4°C until use. MAA Fixation: A 1:1 methanol:acetic acid (MAA) solution was freshly prepared by mixing methanol and glacial acetic acid. Cells were incubated in prechilled MAA at −20°C for 20 min, then washed three times with PBS and stored at 4°C. M2MA Fixation: Cells were first fixed using the M100 protocol, washed three times with PBS, and subjected to immunofluorescence. Following staining, cells were fixed again with MAA (as described above) and stored at 4°C until further use. BE70 Fixation: This fixative was prepared following the MAA protocol and assembled as follows for a 50 mL total volume. A stock solution was prepared using 2.5 mL of 10× PBS (pH 7.4), 1 mL of 50% glycerol, and 0.25 mL of glacial acetic acid. The pH was adjusted to 4.3 by adding 120 μL of 50% NaOH, then diluted to 15 mL with 11.13 mL distilled water. Before use, 15 mL of the stock solution was mixed with 35 mL of absolute ethanol (200 proof). The final composition had a pH of approximately 6.1 and remained free of precipitate when stored at 4°C.

#### Etoposide treatment

U2OS cells were plated in the center of 35 mm dishes containing 14 mm glass-bottom microwell inserts (Cellvis, d35-14-1.5-N) at a density of 100,000 to 150,000 cells per dish on a collagen-coated surface. Cells were allowed to adhere for 20 h at 37°C. Cells were then treated with varying concentrations of etoposide (1 μM–100 μM), diluted in DMEM supplemented with 10% FBS and 1× AA. Etoposide was initially dissolved in DMSO, and control cells were treated with DMSO in DMEM supplemented with 10% FBS and 1× AA. A total of 2 mL media containing either etoposide or control DMSO was added to each dish, and cells were incubated for 2 h at 37°C, 5% CO_2_. Following incubation, cells were fixed with M100 as described above and processed for immunofluorescence analysis.

#### GOLDFISH guide RNA and probe design

For GOLDFISH targeting short genomic regions (<10 kb), all Cas9 binding sites (i.e., PAM sequences) within the region were identified using Benchling. Binding sites were manually selected according to the following criteria: adjacent Cas9 sites were typically spaced 200–400 bp apart, and all guide RNAs annealed to the same strand to ensure directional translocation of Rep-X along the opposite strand. For *ACTB*, the average spacing between Cas9 sites was 327 bp. Oligonucleotide probes targeting the FISH strand were designed using OligoArray 2.1. DNA sequences between Cas9 binding sites were input with the following design parameters: length of 18–30 nt; melting temperature (T_m_) between 72°C and 90°C; GC content between 30% and 70%; maximum T_m_ for predicted secondary structure set to 54°C; minimum T_m_ threshold for cross-hybridization set to 54°C. Probes containing five or more consecutive identical nucleotides were excluded. To further ensure specificity, two additional filters were applied: probes with over 30 off-target binding sites across the human genome were discarded, and those predicted to hybridize with human noncoding RNA or *E. coli* tRNA were also removed. These filtering steps minimized nonspecific interactions with genomic DNA, transcriptome, or *E. coli* tRNA blocking reagents. Probe sequences and sgRNA template DNA are listed in Table S1.

#### sgRNA synthesis, probe design, and probe labeling

All sgRNAs and probes were prepared as previously described.^20^ Designed oligo FISH probes (unlabeled and unmodified) were purchased from IDT and fluorescently labeled according to the published protocol.^20^ Briefly, to conjugate an amino-ddUTP to the 3′ end of each oligonucleotide, 66.7 μM DNA oligonucleotides, 200 μM Amino-11-ddUTP (Lumiprobe, A5040), and 0.4 U/μL Terminal Deoxynucleotidyl Transferase (TdT, Thermo Scientific, EP0162) were combined in 1× TdT Reaction Buffer (Thermo Scientific) and incubated overnight at 37°C. The reaction mixture was purified by ethanol precipitation. Next, the amino-ddUTP-conjugated DNA oligonucleotides were incubated with 100 μg Cy5-NHS (Sigma-Aldrich, GEPA15101) in 0.1 M HEPES buffer (pH 8.5) for 2 h at room temperature, followed by ethanol precipitation. Unlabeled oligonucleotides were removed via high-performance liquid chromatography (HPLC), and the labeled probes were further purified using P4 bead spin columns (Bio-Rad, 1504124).

Single guide RNAs (sgRNAs) targeting the *ACTB* gene were synthesized *in vitro* as a pooled mixture using the EnGen RNA Synthesis Kit (NEB, E3322V), following the manufacturer’s instructions. The resulting RNA was purified using the Monarch RNA Cleanup Kit (NEB, T2040).

#### Purification of Cas9, Cas9-H840A and Rep-X

Cas9 was prepared as previously described.^4^ Cas9-H840A and Rep-X were purified according as previously described.^20^^,^^21^

#### Very fast CRISPR

Guide RNA sequences are listed in Table S1. NPOM-modified caged crRNA was purchased from Biosynthesis. To assemble guide RNA (gRNA), 1.25 μL of 100 μM crRNA was mixed with 1.25 μL of 100 μM tracrRNA (IDT, 1072533) in IDT Duplex Buffer (IDT, 11-01-03-01). The mixture was heated at 95°C for 3 min, then cooled to room temperature for 5 min to allow crRNA/tracrRNA annealing.

To form RNP complexes, 1.5 μL Cas9 (10 μg/μL) (IDT, 1081058) and 1.5 μL dialysis buffer^4^ were mixed with 2 μL of annealed crRNA/tracrRNA. Separately, 16.4 μL of nucleofection solution was mixed with 3.6 μL of supplement (Lonza V4XC1032 for U2OS; Lonza V4XP3032 for RPE1). A total of 1 million cells were collected via 0.05% trypsin, washed with PBS, and resuspended in the supplemented nucleofection solution. RNP complexes and 1 μL of 100 μM Enhancer (IDT) were added to the cells and gently mixed. The mixture was transferred into a Lonza electroporation chamber and electroporated using program CM104 for U2OS cells and EA104 for RPE1 cells on a Lonza 4D X-Unit system. Post-electroporation, cells were transferred into warm medium. Approximately 100,000–150,000 cells were seeded into the central well of a 35 mm dish containing a 14 mm glass-bottom microwell (Cellvis, d35-14-1.5n), pre-coated with collagen (Thermo Scientific, A1048301). Dishes were covered with aluminum foil to protect cells from light and incubated at 37°C with 5% CO_2_ for a minimum of 3 h to allow for complete cell adhesion prior to UV activation. Cas9 was activated via a 30 s UV light pulse using a 365 nm flashlight (Jaxman, B06XW7S1CS). Cells were then fixed at specified time points following activation.

#### Cell cycle synchronization

U2OS cells were synchronized in G1 and S/G2 phases using double and single thymidine block protocols, respectively. Cells were seeded at low confluency into 6-well plates pre-coated with collagen and incubated for a minimum of 12 h prior to synchronization.

Double Thymidine Block (G1 Phase) – Thymidine (2 mM final concentration in DMEM) was added to cells and incubated at 37°C with 5% CO_2_ for 18 h. Cells were then washed twice with PBS and released from the first block by replacing media with fresh DMEM. After 12 h of recovery, cells were washed again with PBS and subjected to a second thymidine block (2 mM final in DMEM) for an additional 18 h. Following synchronization, 1 million cells were collected and electroporated with vfCRISPR targeting *ACTB*. Subsequently, 100,000 cells were plated into 35 mm dishes containing 14 mm glass inserts. Dishes were wrapped in aluminum foil to protect from light and incubated at 37°C, 5% CO_2_ for 15 h. Cas9 cleavage was induced by exposure to UV light.

Single Thymidine Block (S/G2 Phase) – Thymidine (2 mM final in DMEM) was added to cells and incubated at 37°C, 5% CO_2_ for 18 h. Then, 1 million cells were collected via 0.05% trypsinization and electroporated with vfCRISPR targeting *ACTB*. After electroporation, 100,000 cells were plated into 35 mm dishes with 14 mm inserts. Plates were wrapped in aluminum foil and incubated at 37°C, 5% CO_2_ for 6 h. Cas9 cleavage was subsequently induced with UV light exposure.

#### Sanger sequencing

Cells were washed with PBS, collected using 0.05% trypsin/EDTA, and centrifuged at 300 × g for 5 min. A second PBS wash and centrifugation at 300 × g were performed to further clean the sample.

Genomic DNA (gDNA) was extracted using the Qiagen gDNA Extraction Kit (Qiagen, 69504) following the manufacturer’s instructions. Purified gDNA was PCR-amplified using primers targeting the cut site region (Table S1). The thermocycling conditions were as follows:

- Initial denaturation: 98°C for 30 s
- 35 cycles:
- *ACTB*: 98°C for 10 s → 71°C for 30 s → 72°C for 20 s
- *MUC4*: 98°C for 10 s → 69°C for 10 s → 72°C for 20 s
- Final extension: 72°C for 2 min

PCR products were purified using the GeneJet PCR Cleanup Kit (Thermo Fisher Scientific, K0701) according to the manufacturer’s protocol and quantified via Nanodrop. Purified samples were submitted to GENEWIZ (Azenta) for Sanger sequencing. Sequencing data were analyzed using TIDE to assess the frequency of insertion/deletion (indel) events in the population.

#### Immunofluorescence protocol

Cells were plated in the center of 35 mm dishes containing 14 mm glass-bottom microwell inserts (Cellvis, d35-14-1.5-N) at a density of 100,000 to 150,000 cells per dish on a collagen-coated surface. Cells were allowed to adhere for at least 3 h at 37°C. Cells were fixed in ice-cold 100% methanol for 5 min at −20°C, then washed three times with PBS for 5 min each at room temperature. Blocking was performed by incubating cells in 1% BSA (Thermo Fisher Scientific, 37525) diluted in PBS for 1 h at 37°C. Primary antibodies were diluted in 1% BSA blocking buffer and applied to the cells for overnight incubation at 4°C: [53BP1 (Novus, NB100-304SS; 1:2000); BRCA1 (Santa Cruz Biotechnology, sc-6954; 1:500); γH2AX (Abcam, ab26350; 1:1000); H2B (Abcam, ab1790; 1:1000)]. Cells were washed three times with PBS and then incubated for 1 h at room temperature with the following secondary antibodies, diluted 1:2000 in 1% BSA blocking buffer: [Alexa Fluor 488 (Thermo Fisher Scientific, A11001); Alexa Fluor 555 (Abcam, ab150078)]. Afterward, cells were washed three times with PBS for 5 min each at room temperature. Following this, either methanol-acetic acid (MAA) fixation for GOLDFISH was performed, or imaging proceeded directly. Prior to imaging, one drop of Hoechst dye (Invitrogen, R37165) was added to each dish containing 2 mL PBS, and cells were incubated for 5 min at room temperature. Cells were washed three additional times with PBS and imaged in imaging buffer (2× SSC and saturated Trolox (>5 mM), 0.8% (w/v) dextrose) with gloxy (1 mg/mL glucose oxidase, 0.04 mg/mL catalase).

#### GOLDFISH for *ACTB* and *MUC4*

GOLDFISH sgRNA and probe sequences are listed in Table S1. Following immunofluorescence, 2 mL of freshly prepared ice-cold methanol:acetic acid (1:1) was added to the cells and incubated for 20 min at −20°C. Cells were washed three times with PBS for 5 min each at room temperature.

Next, cells were incubated in binding-blocking buffer (20 mM HEPES, pH 7.5, 100 mM KCl, 7 mM MgCl_2_, 5% [v/v] glycerol, 0.1% [v/v] TWEEN 20, 1% [w/v] BSA, freshly added 1 mM DTT, and 0.1 mg/mL *E. coli* tRNA) for 10 min at 37°C. For RNP complex formation, nCas9 and sgRNA were mixed at final concentrations of 440 nM (*ACTB*) or 180 nM (*MUC4*) in binding-blocking buffer and incubated for 30 min at 37°C. The RNP-containing buffer was removed, and blocking binder buffer supplemented with 2 mM ATP and 400 nM Rep-X was added. Cells were incubated for 1.5 h at 37°C in a humidified chamber, followed by three PBS washes. RNase Cocktail Enzyme Mix (Invitrogen, AM2286) was diluted 1:100 in PBS, applied to cells for 30 min at 37°C, and washed three times with PBS. Cells were then incubated for 10 min at 37°C in hybridization buffer (20% [v/v] formamide, 2× SSC, 0.1 mg/mL *E. coli* tRNA, 10% [w/v] dextran sulfate, 2 mg/mL BSA). Fluorescently labeled FISH probes (75 nM for *ACTB*; 83 nM for *MUC4*) diluted in hybridization buffer were applied to cells and incubated for 1.5 h at 37°C. After probe hybridization, cells were washed twice with 30% formamide wash buffer (30% formamide, 2× SSC) for 20 min at 37°C, followed by three PBS washes. Nuclear staining was performed using one drop of Hoechst 33342 Ready Flow Reagent (Invitrogen, R37165) in 2 mL PBS for 5 min at room temperature, then washed three times with PBS. Finally, imaging buffer (2× SSC, saturated Trolox [>5 mM], and 0.8% [w/v] dextrose) supplemented with gloxy (1 mg/mL glucose oxidase and 0.04 mg/mL catalase) was added to the cells for imaging.

#### Fluorescence microscopy

Imaging was performed using a Nikon Eclipse Ti microscope equipped with a Nikon Perfect Focus System and a Xenon arc lamp. The system was controlled via Nikon Elements software. A Nikon 60×/1.49 NA objective (CFI Apo TIRF) was used for image acquisition. Emission was collected through a custom laser-blocking notch filter (ZET488/543/638/750 M; Chroma). Images were captured using an electron-multiplying charge-coupled device (Andor iXon 888) as z-stacks consisting of 20–30 steps with a step size of 300–500 nm.

### Quantification and statistical analysis

Image processing was conducted using Fiji/ImageJ. z stack images were projected into a single plane using the ‘Max Intensity’ Z-Projection function. Image contrast was linearly adjusted by modifying the minimum and maximum values via the ‘Brightness/Contrast’ function for optimal visualization. Cellular parameters were quantified using a custom CellProfiler script. Graphs and statistical analyses were performed using GraphPad Prism. The Kruskal-Wallis test was applied to all cell-level data with a significance threshold of *p* < 0.05. Indel formation scores from Sanger sequencing were calculated using the TIDE software (http://shinyapps.datacurators.nl/tide/).

## Supplemental information

Document S1. Figures S1–S8

Table S1. DNA/RNA oligonucleotide sequences used in vfCRISPR and GOLDFISH experiments, related to Figure 1

Document S2. Article plus supplemental information
