# Supplementary material for: Imaging the time course of DNA damage response at a nonrepetitive endogenous locus
Source: Cell Rep Methods. 2025 Nov 3;5(11):101219. doi: 10.1016/j.crmeth.2025.101219 (PMC12664897; doi:10.1016/j.crmeth.2025.101219)
Supplement: Document S1. Figures S1–S8 [file mmc1.pdf]

Cell Reports Methods, Volume *5*

Supplemental information

Imaging the time course of DNA damage response at a nonrepetitive endogenous locus

Adam T. Rybczynski, W. Taylor Cottle, Po-Ta Chen, Jiwoong Kwon, Tiantian Shang, Yanbo Wang, Paul Meneses, Sushil Pangeni, Yeji Park, Momcilo Gavrilov, and Taekjip Ha

A

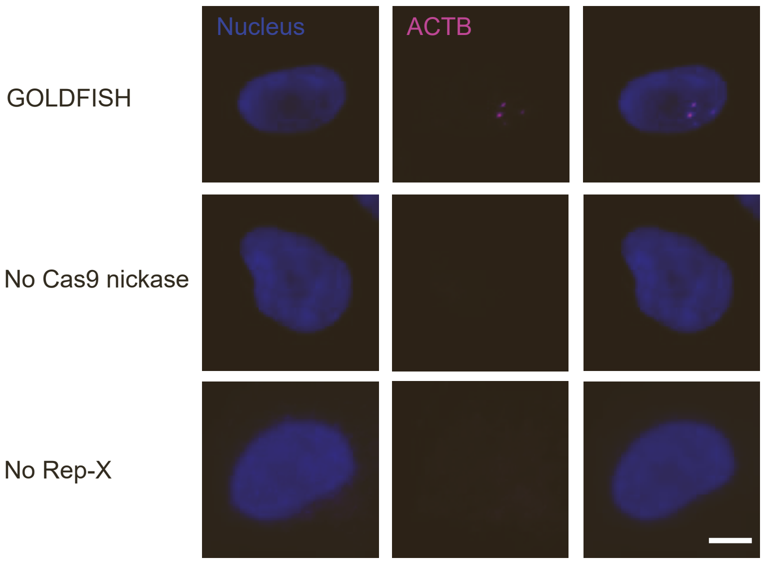

B

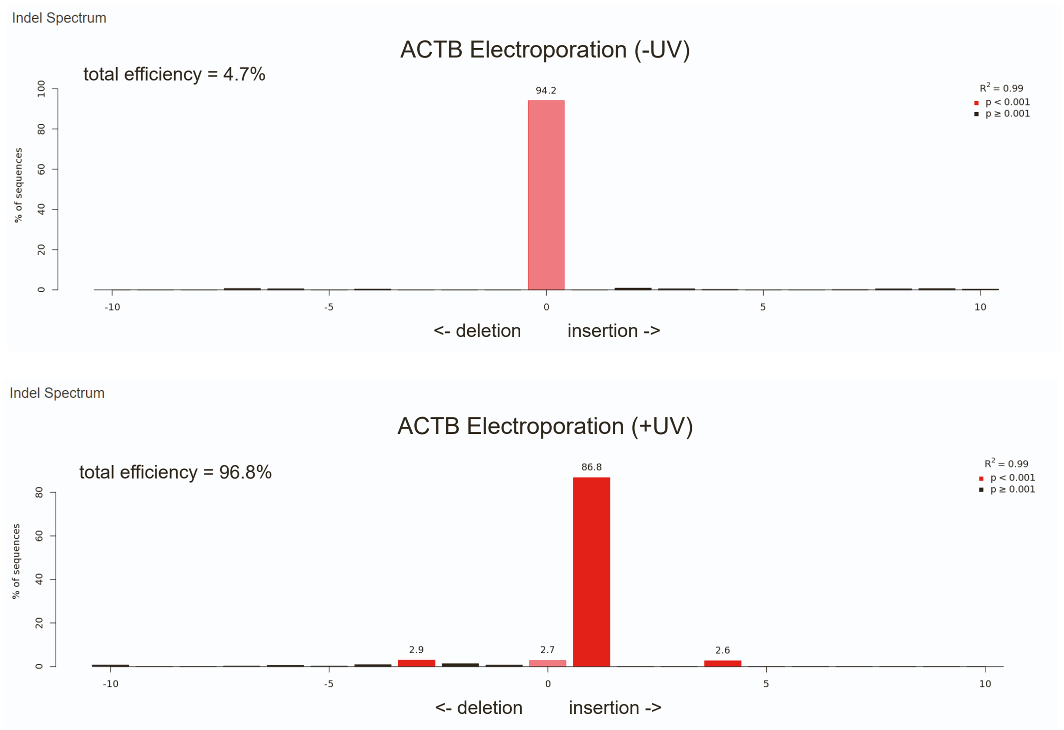

Figure S1. GOLDFISH labeling requires both Cas9 and Rep-X and vfCRISPR cleaves in the presence of UV light, related to Figure 1

(A) Representative images of *ACTB* GOLDFISH (magenta) with nuclear staining by Hoechst (blue). Top panel: *ACTB* GOLDFISH labeling in U2OS cells. Middle panel: *ACTB* GOLDFISH protocol performed without Cas9 nickase. Bottom panel: *ACTB* GOLDFISH protocol performed without Rep-X. Scale bar = 5 μm.

(B) TIDE analysis indicates 4.7% indel formation in U2OS cells electroporated with vfCRISPR targeting *ACTB*. Cells were not exposed to UV light following electroporation (top panel). Samples were collected and subjected to Sanger sequencing 48 hours post-electroporation. TIDE analysis indicates 96.8% indel formation in U2OS cells electroporated with vfCRISPR targeting *ACTB* (bottom panel). Cells were exposed to 30 seconds of UV light three hours after electroporation. Samples were collected and subjected to Sanger sequencing 48 hours post-electroporation.

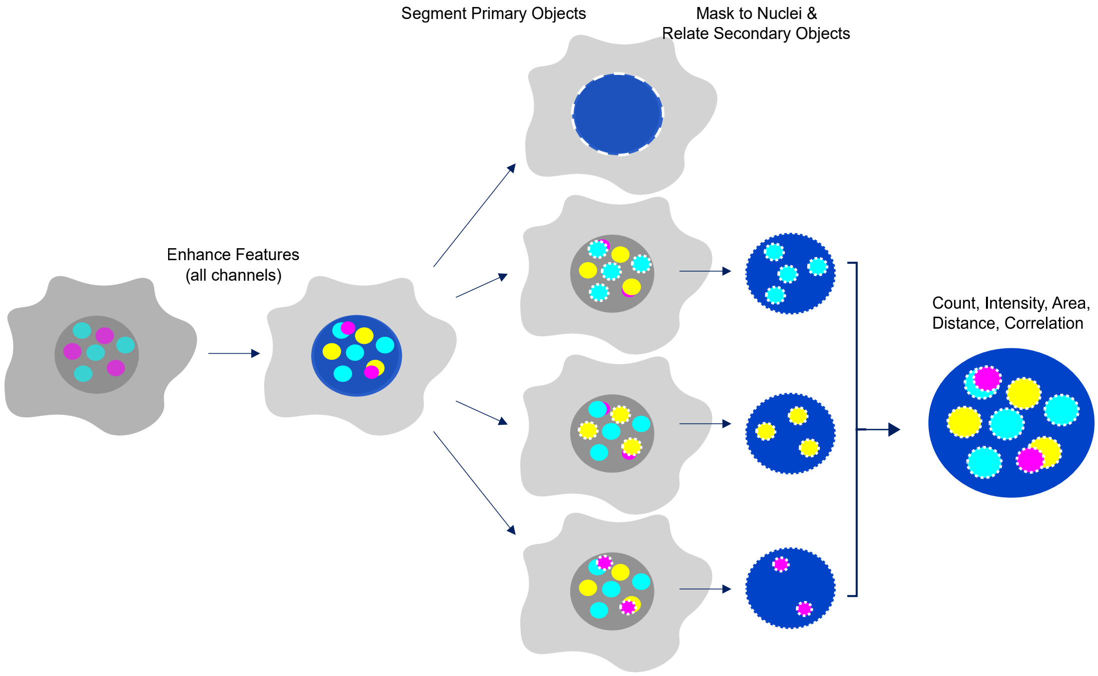

Figure S2. Image analysis of GOLDFISH spots, γH2AX, 53BP1, and BRCA1 foci, related to Figures 1-4
Image analysis of U2OS cells was performed using CellProfiler software to quantify the count, intensity, area, and colocalization of GOLDFISH spots and DNA repair foci. Images were first loaded into CellProfiler, where fluorescence labeling was calibrated and enhanced. Fluorescent signals were then segmented within each cell nucleus, and metrics including signal count, intensity, area, and correlation between overlapping signals across all channels were calculated.

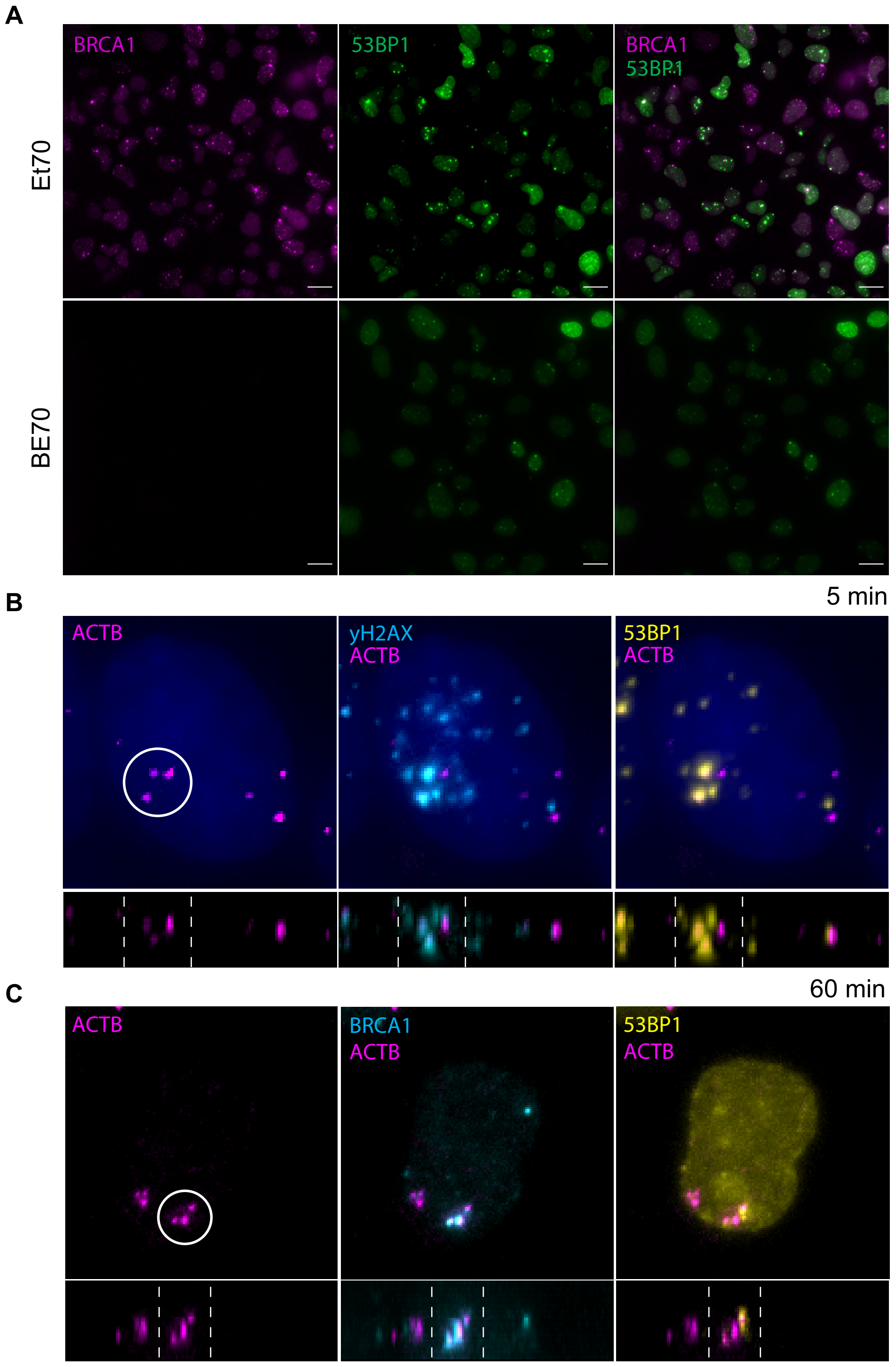

Figure S3. Sequential organic fixation allows for the pairing of IF and GOLDFISH, related to Figure 2

(A) Representative immunofluorescent (IF) images of BRCA1 (magenta) and 53BP1 (green) after fixation with 70% Ethanol (Et70, top panel) or buffered 70% Ethanol, containing Acetic Acid (BE70, bottom panel). U2OS cells were not electroporated with vfCRISPR.

(B) Representative *ACTB* GOLDFISH (magenta) with γH2AX (cyan) and 53BP1 (yellow) after sequential organic fixation (methanol fixation → IF→ methanol acetic acid → GOLDFISH) to pair IF and GOLDFISH. U2OS cells fixed 5 mins post-vfCRISPR activation at *ACTB*.

(C) Representative *ACTB* GOLDFISH (magenta) with BRCA1 (cyan) and 53BP1 (yellow) after sequential organic fixation (methanol fixation → IF → methanol acetic acid → GOLDFISH) to pair IF and GOLDFISH. U2OS cells fixed 60 mins post-vfCRISPR activation at *ACTB*.

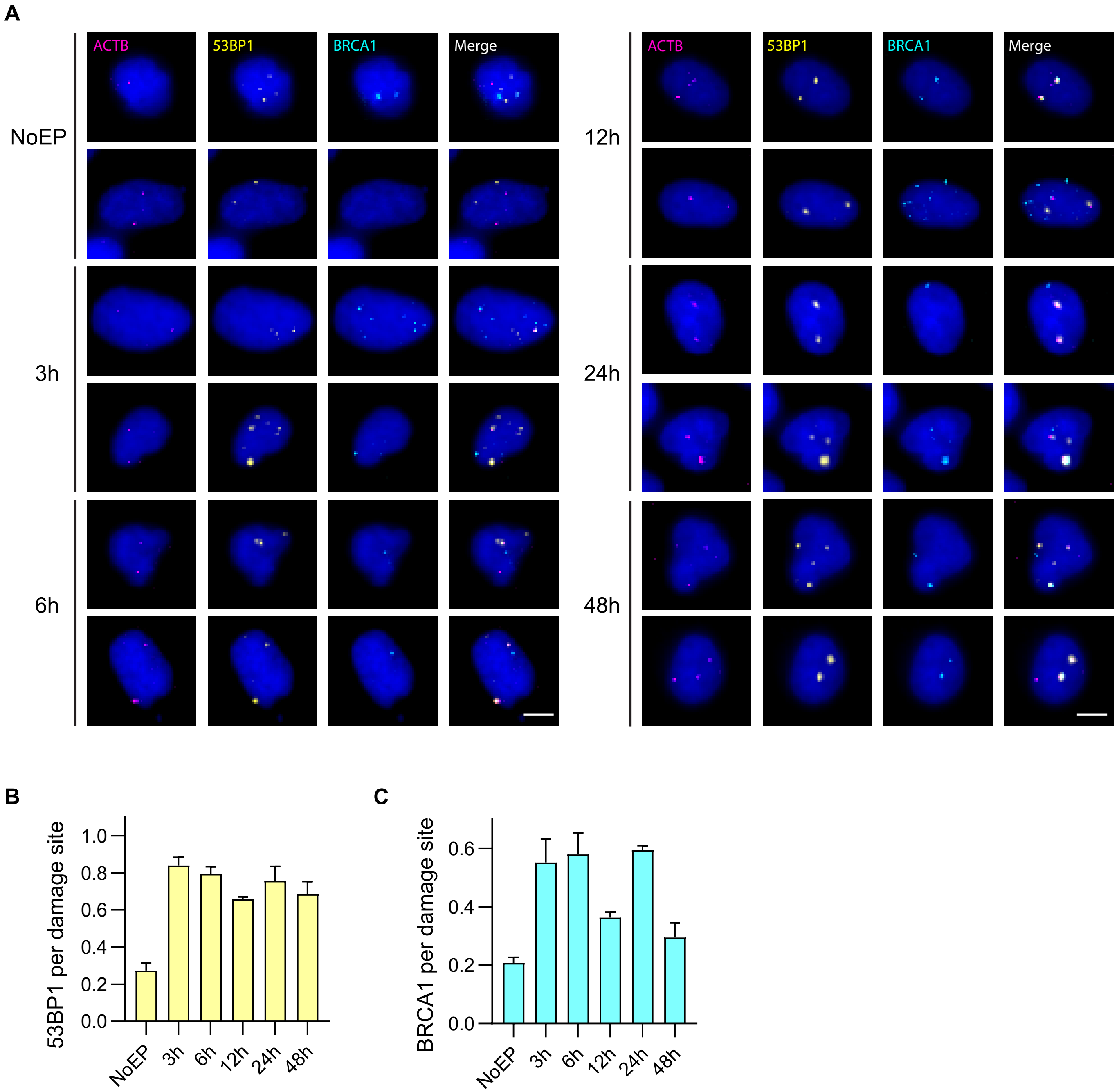

Figure S4. Time-course analysis of 53BP1 and BRCA1 recruitment to *ACTB* damage following electroporation in U2OS cells, related to Figure 3

(A) Representative images of U2OS cells without electroporation (NoEP) and cells electroporated with CRISPR-Cas9 with regular gRNA targeting *ACTB*. Cells were fixed at 3 hours (3h), 6 hours (6h), 12 hours (12h), 24 hours (24h), and 48 hours (48h) post-electroporation.

(B) Proportion of U2OS cells with colocalized 53BP1 foci and *ACTB* spots among cells containing both markers, measured over time following CRISPR-Cas9 electroporation. NoEP = 0.27; 3h = 0.83; 6h = 0.79; 12h = 0.66; 24h = 0.76; 48h = 0.69.

(C) Proportion of U2OS cells with colocalized BRCA1 foci and *ACTB* spots among cells containing both markers, measured over the same time course. NoEP = 0.2; 3h = 0.55; 6h = 0.58; 12h = 0.36; 24h = 0.59; 48h = 0.30.

All data are presented as mean ± SEM (number of replicates = 2, ~30 cells per condition per replicate).

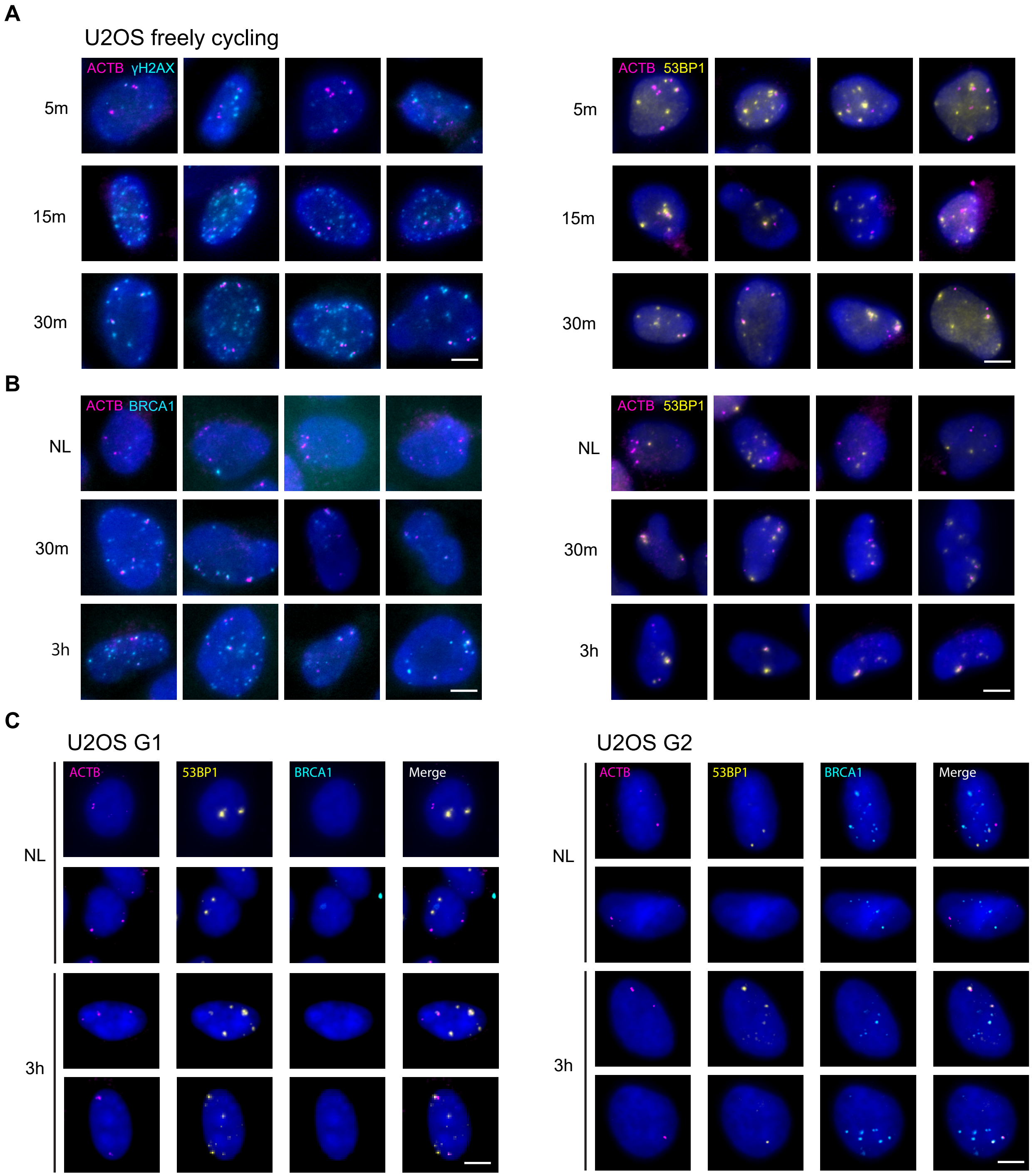

Figure S5. Additional time-course images of DNA repair foci formation at *ACTB* after vfCRISPR-induced *ACTB* damage in U2OS cells, related to Figures 3-4

(A) Representative images of freely cycling U2OS cells stained for γH2AX (cyan) and 53BP1 (yellow) at *ACTB* loci (magenta) at three time points following UV light exposure and subsequent vfCRISPR activation at *ACTB*: 5 minutes (5m), 15 minutes (15m), and 30 minutes (30m). Scale bar = 5 μm.

(B) Representative images of freely cycling U2OS cells showing BRCA1 (cyan) and 53BP1 (yellow) colocalized at *ACTB* (magenta) in cells not exposed to UV light (NL), and cells fixed at 30 minutes (30m) and 3 hours (3h) after UV light. Scale bar = 5 μm.

(C) Cells synchronized in G1 and G2 phases were electroporated with vfCRISPR targeting *ACTB*. A control group was not shown UV light (No Light - NL), and cells that were exposed to UV light were fixed 3 hours after UV exposure. Fixed cells then underwent immunofluorescence for 53BP1 (yellow) and BRCA1 (cyan), followed by GOLDFISH at *ACTB* (magenta). Scale bar = 5 μm.

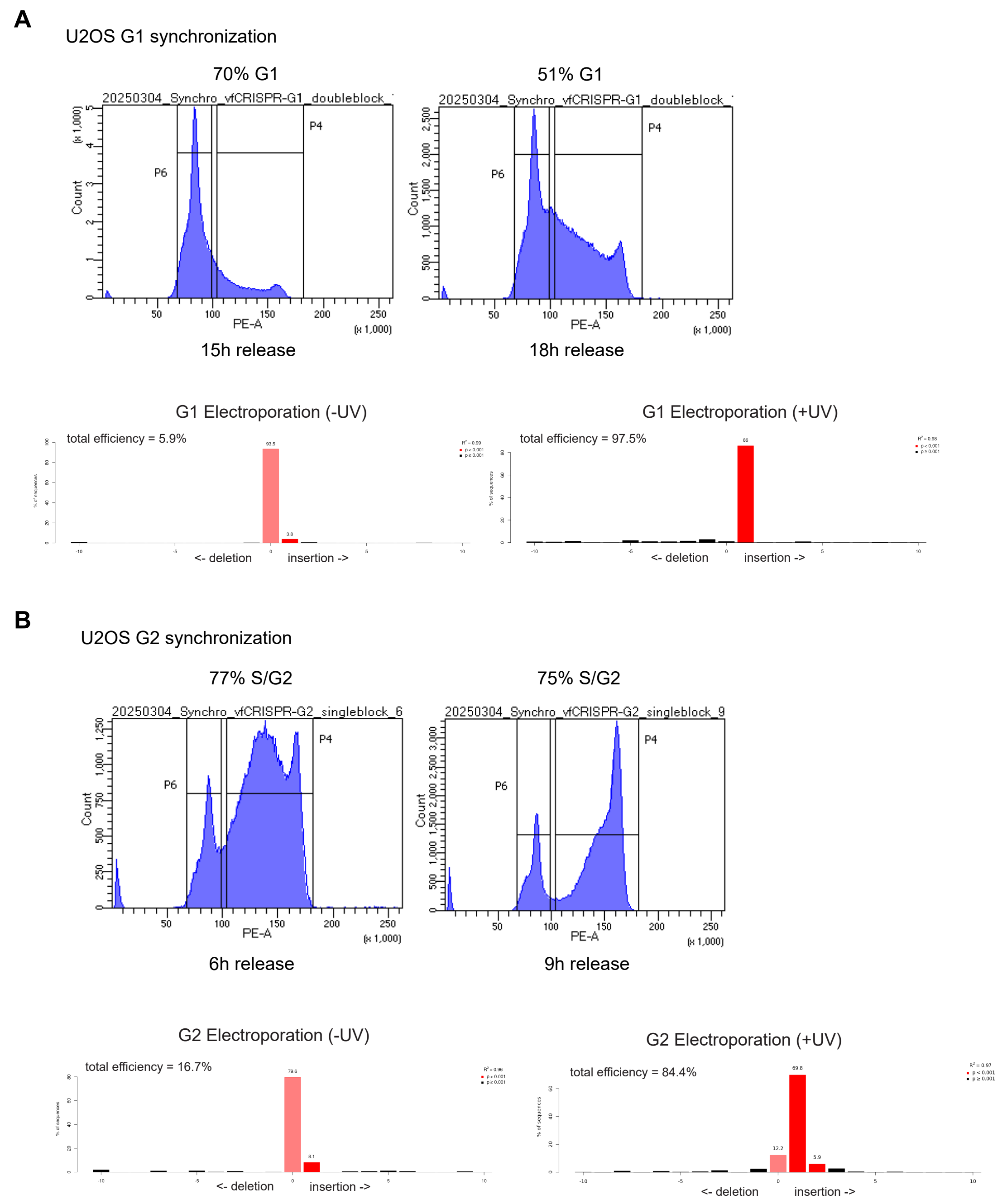

Figure S6. Cell cycle phase-specific vfCRISPR targeting of *ACTB* in synchronized U2OS cells, related to Figure 4

(A) G1-phase synchronization using double thymidine block. U2OS cells were electroporated with vfCRISPR targeting *ACTB* following release from the second block. Cells were exposed to UV light 15 hours post-release, with 70% of cells in G1 phase at the time of vfCRISPR activation. Fixation occurred at 18 hours post-release, with 51% of cells in G1 phase. A subset of vfCRISPR-activated cells was collected 48 hours later and analyzed by Sanger sequencing, revealing 97.5% indel efficiency at *ACTB*. Non-activated control cells (NL) were collected concurrently and showed 5.9% indel formation.

(B) S/G2-phase synchronization using single thymidine block. Cells were electroporated with vfCRISPR targeting *ACTB* after release. UV exposure occurred 6 hours post-release, when 77% of cells were in S/G2 phase. Cells were fixed at 9 hours post-release, with 75% in S/G2 phase. A subset of vfCRISPR-activated cells was collected 48 hours later and Sanger sequenced, showing 84.4% indel efficiency. Non-activated control cells (NL) collected at the same time demonstrated 16.7% indel formation.

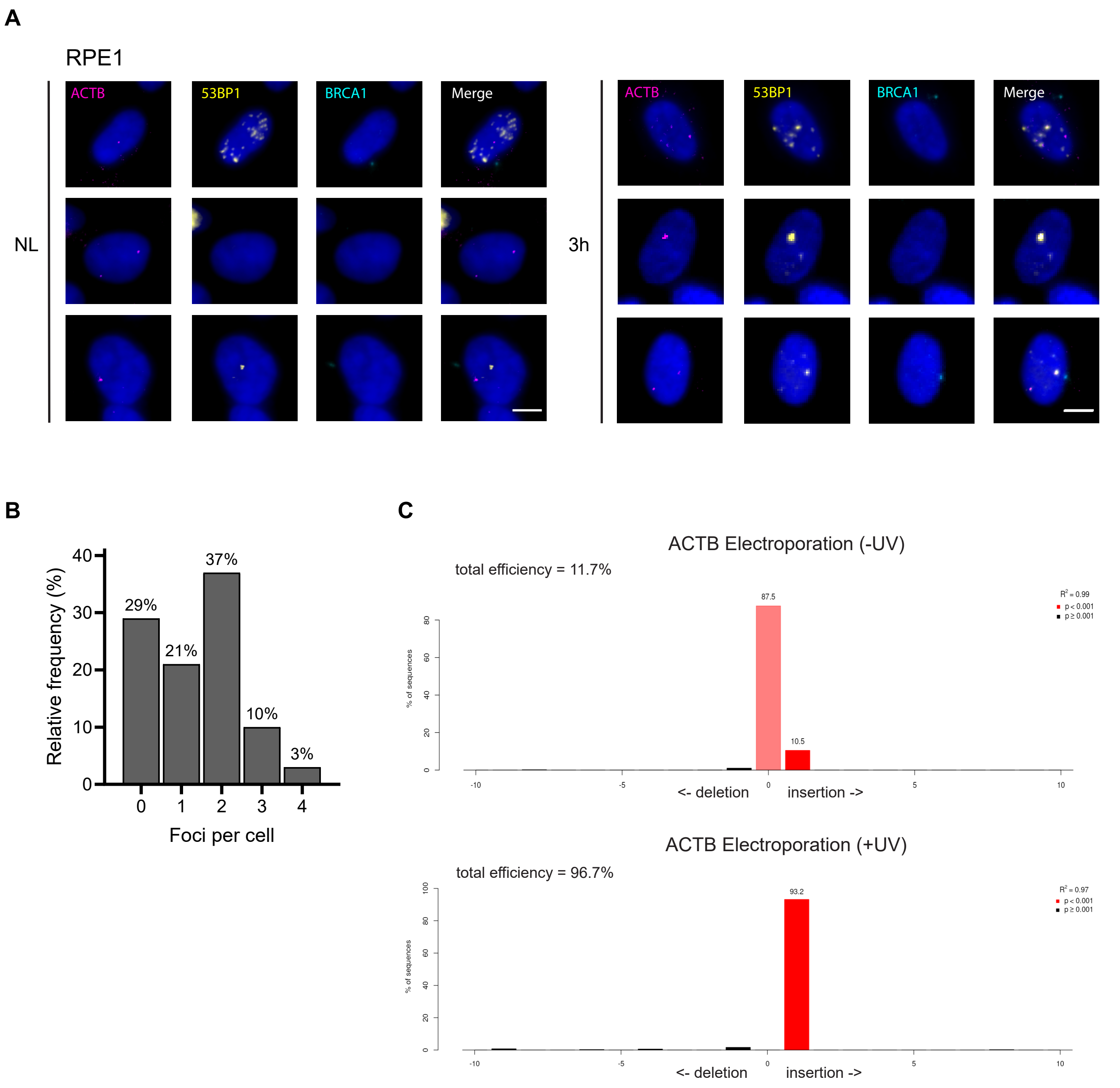

Figure S7. Additional images of repair foci formation in RPE1 cells with vfCRISPR electroporation targeting *ACTB*, related to Figure 4

(A) RPE1 cells were electroporated with vfCRISPR targeting *ACTB*. A control group was not exposed to UV light (No Light – NL) and the cells that were exposed to UV light were fixed 3 hours after UV (3h). Fixed cells then underwent immunofluorescence for 53BP1 (yellow) and BRCA1 (cyan), followed by GOLDFISH at *ACTB* (magenta). Scale bar = 5 μm.

(B) Histogram of the number of GOLDFISH *ACTB* foci count per RPE1 cell (n = 2, ~100 cells/replicate).

(C) RPE1 cells that were electroporated with vfCRISPR targeting *ACTB* were not exposed to UV light (-UV) or exposed to UV light (+UV), collected 48 hours after UV dose, and Sanger sequenced. TIDE analysis showed 11.7% indel formation at *ACTB* for the RPE1 cells not exposed to UV light and 96.7% indel formation for RPE1 cells exposed to UV light.

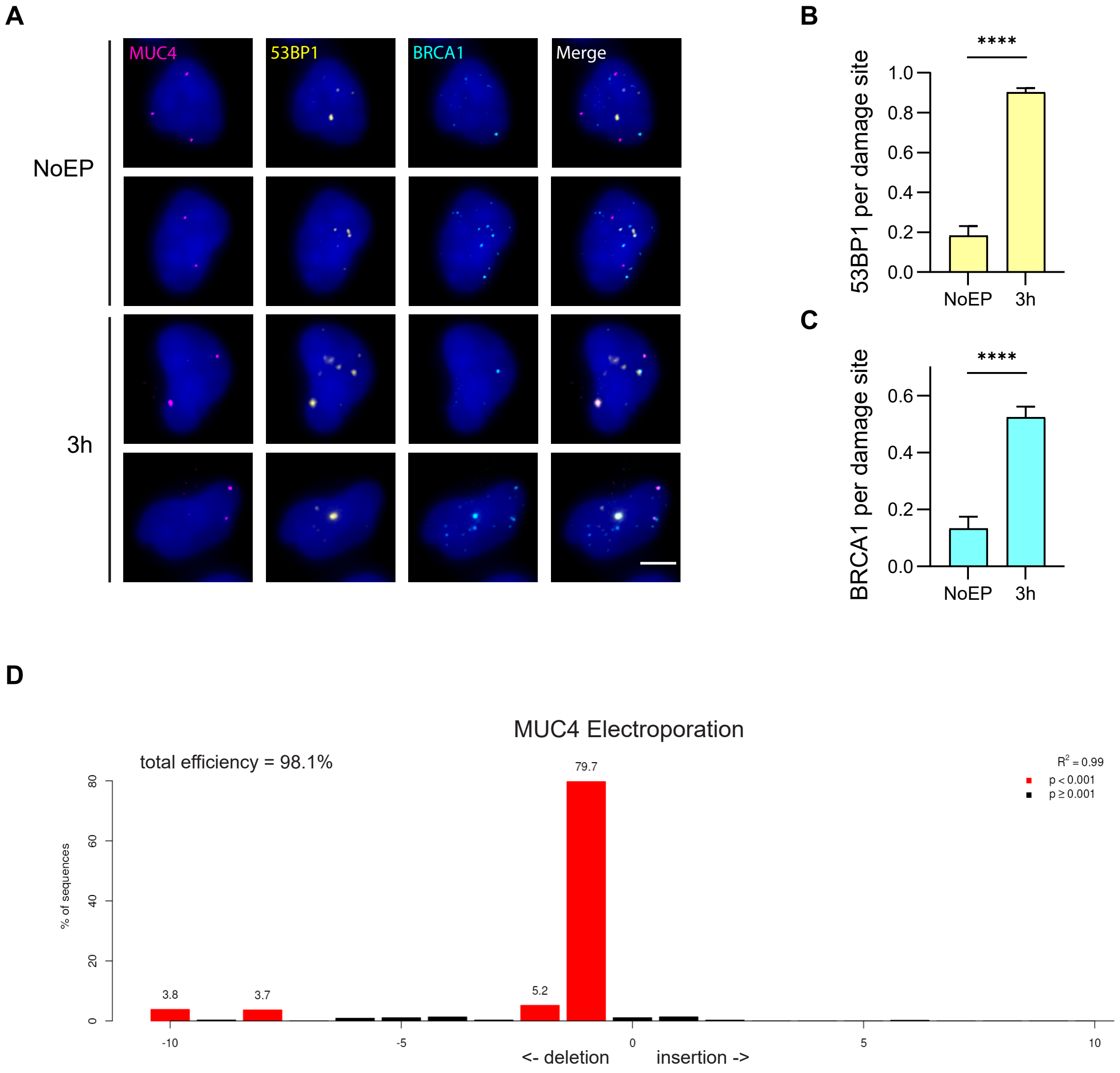

Figure S8. Recruitment kinetics of 53BP1 and BRCA1 at the endogenous nonrepetitive locus *MUC4*, related to Figure 4

(A) Representative images of U2OS cells either not electroporated (NoEP) or electroporated with CRISPR-Cas9 targeting *MUC4*, then fixed 3 hours post-electroporation (3h). All samples underwent immunofluorescence staining for 53BP1 (yellow) and BRCA1 (cyan), followed by GOLDFISH targeting *MUC4* (magenta). Scale bar = 5 µm.

(B) Proportion of U2OS cells exhibiting colocalization of 53BP1 foci with *MUC4* among those with both 53BP1 and *MUC4* foci, shown as a function of time post-CRISPR-Cas9 electroporation. NoEP = 0.18; 3h = 0.90.

(C) Proportion of U2OS cells exhibiting colocalization of BRCA1 foci with *MUC4* among those with both BRCA1 and *MUC4* foci, shown as a function of time post-CRISPR-Cas9 electroporation. NoEP = 0.13; 3h = 0.52.

All data are presented as mean ± SEM. Statistical significance was determined using the Kruskal–Wallis test; **** = $p < 0.0001$ (number of replicates = 2, ~30 cells per condition per replicate).

(D) U2OS cells electroporated with CRISPR-Cas9 targeting *MUC4* were collected 48 hours post-electroporation and analyzed via Sanger sequencing. TIDE analysis revealed an indel efficiency of 98.1% at *MUC4*.
